# Supplementary material for: Glucocerebrosidase is imported into mitochondria and preserves complex I integrity and energy metabolism
Source: Nat Commun. 2023 Apr 6;14:1930. doi: 10.1038/s41467-023-37454-4 (PMC10079970; doi:10.1038/s41467-023-37454-4)
Supplement: Supplementary file 3 — Description of Additional Supplementary Files [file 41467_2023_37454_MOESM3_ESM.pdf]

Description of Additional Supplementary Files

File Name: Supplementary Dataset 1

List of GCase interactors from quantitative proteomic analysis.

File Name: Supplementary Dataset 2

List of differentially expressed proteins from quantitative proteomic analysis.

File Name: Supplementary Dataset 3

scRNAseq dataset of in *GBA1* mutant midbrain organoids and isogenic controls.

File Name: Supplementary Dataset 4

List of Differentially Expressed Genes (DEGs) in *GBA1* mutant midbrain organoids and isogenic controls.
