# Supplementary material for: Glucocerebrosidase is imported into mitochondria and preserves complex I integrity and energy metabolism
Source: Nat Commun. 2023 Apr 6;14:1930. doi: 10.1038/s41467-023-37454-4 (PMC10079970; doi:10.1038/s41467-023-37454-4)
Supplement: Supplementary file 8 — Reporting Summary [file 41467_2023_37454_MOESM8_ESM.pdf]

nature portfolio

Corresponding author(s): Michela Deleidi

Last updated by author(s): 2023.03.06

Reporting Summary

Nature Portfolio wishes to improve the reproducibility of the work that we publish. This form provides structure for consistency and transparency in reporting. For further information on Nature Portfolio policies, see our Editorial Policies and the Editorial Policy Checklist.

Statistics

For all statistical analyses, confirm that the following items are present in the figure legend, table legend, main text, or Methods section.

n/a	Confirmed	
☐	☒	The exact sample size (*n*) for each experimental group/condition, given as a discrete number and unit of measurement
☐	☒	A statement on whether measurements were taken from distinct samples or whether the same sample was measured repeatedly
☐	☒	The statistical test(s) used AND whether they are one- or two-sided<br>*Only common tests should be described solely by name; describe more complex techniques in the Methods section.*
☒	☐	A description of all covariates tested
☐	☒	A description of any assumptions or corrections, such as tests of normality and adjustment for multiple comparisons
☐	☒	A full description of the statistical parameters including central tendency (e.g. means) or other basic estimates (e.g. regression coefficient) AND variation (e.g. standard deviation) or associated estimates of uncertainty (e.g. confidence intervals)
☐	☒	For null hypothesis testing, the test statistic (e.g. *F*, *t*, *r*) with confidence intervals, effect sizes, degrees of freedom and *P* value noted<br>*Give P values as exact values whenever suitable.*
☒	☐	For Bayesian analysis, information on the choice of priors and Markov chain Monte Carlo settings
☒	☐	For hierarchical and complex designs, identification of the appropriate level for tests and full reporting of outcomes
☐	☒	Estimates of effect sizes (e.g. Cohen's *d*, Pearson's *r*), indicating how they were calculated

Our web collection on statistics for biologists contains articles on many of the points above.

Software and code

Policy information about availability of computer code

Data collection	Images were acquired with a Leica TCS SP8 confocal microscope instrument. Image J was used for quantification. Oxygen consumption ratewas analyzed using an XFp or XFe96 Extracellular Flux Analyzer (Seahorse Biosciences). For the assessment of the iMTS-Is, the mTP score was assesed with TargetP. Orbitrap Fusion™ Lumos™ Tribrid™ Mass Spectrometer (Thermo Fisher Scientific) was used to aquire proteomic data. scRNA-seq libraries were generated using the 10X Chromium Next GEM Single Cell 3' Reagent Kit v3.1 according to the manufacturer's instructions and paired-end sequenced on an Illumina NovaSeq 6000 (SP Flow Cell).
Data analysis	GraphPad Prism version 9.3.1 was used for statistical analysis. For analysis of the oxygen consumption rate, the Seahorse Wave (Version 2.6) was used. For image acquisition the Zen Blue (Zeiss) and the Leica Las X software (Version 5.1.0) were used. Mass spectrometry data were analysed with IsobarQuant, Mascot (v2.2.07), Panther, Human Mitocarta 3.0 database, Cytoscape (version 3.9.0) and GeneCards. IFF/IHC image analysis and densitometric analysis of Western blots were performed with Fiji-ImageJ version 2.3.0/1.53q. For scRNAseq analysis, publicly available versions of R (4.1.0), Seurat R package (4.1.0), and Monocle3 (1.0.0) were used. Scripts, packages, and R session info used in this study are available at https://doi.org/10.5281/zenodo.6475864.

For manuscripts utilizing custom algorithms or software that are central to the research but not yet described in published literature, software must be made available to editors and reviewers. We strongly encourage code deposition in a community repository (e.g. GitHub). See the Nature Portfolio guidelines for submitting code & software for further information.

Data

Policy information about availability of data

All manuscripts must include a data availability statement. This statement should provide the following information, where applicable:

- Accession codes, unique identifiers, or web links for publicly available datasets
- A description of any restrictions on data availability
- For clinical datasets or third party data, please ensure that the statement adheres to our policy

The main data supporting the findings of this study are available within the article and its Supplementary files. The mass spectrometry proteomics data have been deposited in ProteomeXchange via the PRIDE partner repository under the accession code PXD032155. For scRNAseq data GEO accession number GSE198033. Protocols have been deposited at https://dx.doi.org/10.17504/protocols.io.8epv593dng1b/v1. The UniProt Homo sapiens database (UP000005640, https://www.uniprot.org/proteomes/UP000005640) was used as the reference proteome. Human Mitochondrial proteins were identified according to the Human Mitocarta 3.0 database (https://www.broadinstitute.org/files/shared/metabolism/mitocarta/human.mitocarta3.0.html; RRID:SCR_004869). The subcellular localization of the top 100 candidate proteins was assigned according to the GeneCards database version 5.6 (https://www.genecards.org; RRID:SCR_002773) by using a confidence level 4.

Human research participants

Policy information about studies involving human research participants and Sex and Gender in Research.

Reporting on sex and gender	Sex and gender were not considered in the study design.
Population characteristics	There is no population characteristic analysis in the present manuscript
Recruitment	Human skin fibroblast were collected from individuals with GBA1 mutations.
Ethics oversight	Skin fibroblasts from all patients were obtained with informed consent approved by the Ethics Committee of the Medical Faculty and the University Hospital Tübingen.

Note that full information on the approval of the study protocol must also be provided in the manuscript.

Field-specific reporting

Please select the one below that is the best fit for your research. If you are not sure, read the appropriate sections before making your selection.

☒ Life sciences ☐ Behavioural & social sciences ☐ Ecological, evolutionary & environmental sciences

For a reference copy of the document with all sections, see nature.com/documents/nr-reporting-summary-flat.pdf

Life sciences study design

All studies must disclose on these points even when the disclosure is negative.

Sample size	Sample size determination is based on the previous experience to obtain significance and reproducibility. No statistical methods were used to predetermine sample sizes. All data resulted from at least 3 independent differentiation onsets with at least 3 technical replicates. The sample sizes were sufficient for statistical analysis and are listed in each figure legend.
Data exclusions	No data were excluded from the analyses.
Replication	All attempts at replication were successful. All data resulted from at least 3 independent experiments, the number of independent experiments is indicated in the figure legends.
Randomization	NA. Cells were grouped based in the genotype.
Blinding	Investigators were blinded to the groups and samples. Investigators involved in data collection of scRNAseq and proteomic data were blinded, investigators performing data analysis were not blinded. The investigators were not blinded because no bias could be made in the analysis, raw data provided for data analysis is publicly available.

Reporting for specific materials, systems and methods

We require information from authors about some types of materials, experimental systems and methods used in many studies. Here, indicate whether each material, system or method listed is relevant to your study. If you are not sure if a list item applies to your research, read the appropriate section before selecting a response.

Materials & experimental systems

n/a	Involved in the study
	☒ Antibodies
	☒ Eukaryotic cell lines
☒	☐ Palaeontology and archaeology
☒	☐ Animals and other organisms
☒	☐ Clinical data
☒	☐ Dual use research of concern

Methods

n/a	Involved in the study
☒	☐ ChIP-seq
☒	☐ Flow cytometry
☒	☐ MRI-based neuroimaging

Antibodies

Antibodies used

IP:
Rabbit anti-GCase MaxPab (10 µg, Abnova, H00002629-D01)
Rabbit anti DYKDDDDK Tag (Flag) (1:1000, Cell Signalling Technology, 14793S)
Mouse anti-HSP60 (1:3000, Santa Cruz Biotechnology, sc-271215)
Mouse anti-LIMP2, 1:1000, Santa Cruz, Sc-55571)
Rabbit anti-LONP1(1:2000, Proteintech, 15440-1-AP)
Mouse anti-LONP1 (1:5000, Proteintech, 66043-1-lg)
Mouse anti-NDUFS2 (1:250, Santa Cruz Biotechnology, sc-390596)
Mouse anti-TIM23 (1:1000, BD Bioscience, 611222)
Mouse anti-TIMMDC1 (1:100, Santa Cruz, sc-514926)
Normal Rabbit IgG (10 µg, Covalab, pab01004-P)

Western blot:
Mouse anti-α-Synuclein (1:1000, BD Bioscience, 610787)
Rabbit anti-α-Synuclein (1:1000, Millipore, 04-1053)
Mouse anti-ATP5A (1:2000, Abcam, ab14748)
Mouse anti-ATP5B (1:1000, Santa Cruz Biotechnology, sc-135903)
Mouse anti-ß-Actin (1:5000, Santa Cruz Biotechnology, sc-47778)
Mouse anti-Calnexin (TO-5) (1:200, Santa Cruz Biotechnology, sc-80645)
Mouse anti DJ-1 (1:1000, Santa Cruz Biotechnology, sc-55572)
Rabbit anti DYKDDDDK Tag (Flag) (1:1000, Cell Signalling Technology, 14793S)
Mouse anti-GAPDH (1:10000, Santa Cruz Biotechnology, sc-47724)
Mouse anti-GCase (1:1000, Sigma Aldrich, WM0002629M1)
Rabbit anti-GRP78 (1:8000, Proteintech, 11587-1-AP)
Rabbit anti-GRP94 (1:1000, Proteintech,14700-1-AP)
Mouse anti-HEXB (1:1000, Santa Cruz, Sc-376781)
Rabbit anti-HSC70/HSPA8 (1:1000, Cell Signaling Technology, 8444S)
Mouse anti-HSC70 (1:1000, Santa Cruz Biotechnology, sc-7298)
Mouse anti-HSP60 (1:3000, Santa Cruz Biotechnology, sc-271215)
Mouse anti-LAMP1 (1:100, DSHB, H4A3)
Mouse anti-LIMP2, 1:1000, Santa Cruz, Sc-55571)
Rabbit anti-LIMP2 (1:500, ProSci, 4621)
Rabbit anti-LONP1(1:2000, Proteintech, 15440-1-AP)
Mouse anti-LONP1 (1:5000, Proteintech, 66043-1-lg)
Mouse anti-NDUFA9 (10000, Abcam, ab14713)
Mouse anti-NDUFA10 (1:4000, Santa Cruz, sc-376357)
Rabbit anti-NDUFS1 (1:1000, GeneTex, GTX113787)
Mouse anti-NDUFS2 (1:250, Santa Cruz Biotechnology, sc-390596)
Mouse anti-SDHA (1:10000, Abcam, Ab14715)
Rabbit anti-TBC1D15 (1:2500, Novus Biologicals, NBP2-36552)
Mouse anti-TIMMDC1 (1:1000, Santa Cruz, sc-514927)
Mouse anti-TIMMDC1 (1:100, Santa Cruz, sc-514926)
Rabbit anti-TIMMDC1 (1:1000, Atlas Antibodies-Sigma, HPA053214)
Rabbit anti-TIM23 (1:1000, Proteintech,11123-1-AP)
Mouse anti-TIM23 (1:1000, BD Bioscience, 611222)
Mouse anti-TOM20 (1:1000, Santa Cruz Biotechnology, sc-11415)
Rabbit anti-TOM70 (1:1000, ProteinTech, 14528-1-AP
Rabbit anti-Tyrosine Hydroxylase (1:1000, Pel-Freez Biologicals, P40101-150)
Mouse anti-Vimentin (1:2000, BD Bioscience, 550513)
Mouse anti-Vinculin (1:1000, Santa Cruz, sc-73614)

Western Blot secondary antibodies:
appropriate species of HRP-conjugated secondary antibody (1:5000, Cell Signaling Technology, 7076S Mouse - 7074S Rabbit)
appropriate species of fluorescence secondary antibody (1:10000, LI-COR IRDye®, 680 RD Goat anti Mouse- 800CW Goat anti-Rabbit)

BNE:
Mouse anti-NDUFA9 (1:5000, Abcam, ab14713)
Mouse anti-SDHA (1:5000, Abcam, Ab14715)
Rabbit anti-TIMMDC1 (1:1000, Atlas Antibodies-Sigma, HPA053214)

Immunocytochemistry primary antibodies:
Mouse anti-α-Synuclein (1:250, BD Bioscience, 610787)
Mouse anti-α-Synuclein Phospho Ser129 (1:500, Biolegend, 825701)
Rabbit anti Dopa Decarboxylase (1:250, Thermo Fisher, PA5-25450)
Rabbit anti Dopamine Transporter (1:100, Proteintech, 22524-1-AP)
Rabbit anti DYKDDDDK Tag (Flag) (1:1000, Cell Signalling Technology, 14793S)
Goat anti-FOXA2 (1:100, Santa Cruz Biotechnology, Sc-6553)
Mouse anti-FOXA2 (1:100, Santa Cruz Biotechnology, Sc-101060)
Rabbit anti-GCase (1:100, Proteintech, 20622-1-AP)
Mouse anti-GFP (1:500, Sigma Aldrich, G6539)
Rabbit anti-HSP60 (1:2000, Cell Signalling Technology,12165)
Mouse anti-HSP60 (1:2000, Santa Cruz Biotechnology, sc-271215)
Mouse anti- LONP1 (1:100, Proteintech, 66043-1-lg)
Chicken anti- MAP2 (1:2500, Biolegend, 82250)
Goat anti-OTX2 (1:200, Neuromics, GT15095)
Mouse anti-S100B (1:500, Sigma-Aldrich, S2532)
Mouse anti-SOX2 (1:200, Abcam, Ab75485)
Mouse anti-Synaptophysin (1:500, Proteintech, CL488-67864)
Mouse anti-ß3-Tubulin (1:1000, Biolegend, 801202)
Rabbit anti-ß-Tubulin (1:1000, Biolegend E104092F)
Mouse anti-TOM20 (1:500, Santa Cruz Biotechnology, sc-11415)
Rabbit anti-Tyrosine Hydroxylase (1:500, Pel-Freez Biologicals, P40101-150)

Immunocytochemistry secondary antibodies:
appropriate species of Alexa Fluor488/568/647-conjugated secondary antibody (1:1000, Invitrogen)

Validation

All antibodies used in this study have been commercially validated, information is available on the manufacturer´s websites. GCase antibodies were validated in KO human cell lines or tissues via immunoblotting or IHC/IF prior to experimentation.

Eukaryotic cell lines

Policy information about cell lines and Sex and Gender in Research

Cell line source(s)

All iPSCs used in the study were derived from patients who signed an informed consent . The Ethics Committee of the Medical Faculty and the University Hospital Tübingen (Ethikkommission der Medizinischen Fakultät am Universitätsklinikum Tübingen) approved the protocol before performing the experiments.
Flp-In™ 293 T-Rex cells are commercially available (Thermo Fisher Scientific, # R78007)

Authentication

iPSC Cell lines were routinely assessed by Sanger sequencing for cell line identity. Flp-In™ 293 T-Rex cells were kept in antibody selection to confirm integration of the expression vectors.

Mycoplasma contamination

All cell lines were routinely assessed for mycoplasma using a Venor®GeM Classic kit (Minerva Biolabs). All cell lines tested negative for mycoplasma contamination.

Commonly misidentified lines
(See ICLAC register)

There are no commonly misidentified cell lines.
