# Supplementary material for: Glucocerebrosidase is imported into mitochondria and preserves complex I integrity and energy metabolism
Source: Nat Commun. 2023 Apr 6;14:1930. doi: 10.1038/s41467-023-37454-4 (PMC10079970; doi:10.1038/s41467-023-37454-4)
Supplement: Supplementary file 1 — Supplementary Information [file 41467_2023_37454_MOESM1_ESM.pdf]

SUPPLEMENTARY INFORMATION

Glucocerebrosidase, a Parkinson´s disease-associated protein, is imported into mitochondria and preserves complex I integrity and energy metabolism

Pascale Baden, Maria Jose Perez, Hariam Raji, Federico Bertoli, Stefanie Kalb, María Illescas, Fokion Spanos, Claudio Giuliano, Alessandra Maria Calogero, Marvin Oldrati, Hannah Hebestreit, Graziella Cappelletti, Kathrin Brockmann, Thomas Gasser, Anthony H V Schapira, Cristina Ugalde, Michela Deleidi

*Manuscript correspondence:
Michela Deleidi; michela.deleidi@institutimagine.org

Supplementary Table 1. Human iPSC lines used in the study.

# Parental line	Gender	Diagnosis	Age at Sampling	Age of onset	Genotype	iPSC clones	Isogenic pairs and corresponding genotype
L444P/WT-1	M	PD	60	49	L444P/WT	L444P/WT-1<br>L444P/WT-2	L444P/WT-1 GC (WT/WT)<br>L444P/L444P-1 (L444P/L444P)*
L444P/L444P-2 [1]	F	GD type 3	2	2	L444P/L444P	L444P/L444P-2A<br>L444P/L444P-2B	L444P/L444P-2A GC1 [2] (WT/WT)<br>L444P/L444P-2A GC2 [2] (WT/WT)
E326K/WT-1	M	PD	62	52	E326K/WT	E326K/WT-1A<br>E326K/WT-1B	-
E326K/WT-2	M	PD	60	50	E326K/WT	E326K/WT-2A<br>E326K/WT-2B	E326K/WT-2A GC1 [3] (WT/WT)<br>E326K/WT-2A GC2 [3] (WT/WT)
GBA1 knockout [4]	F	HC	62	/	GBA1 -/-	/	/

PD, Parkinson's disease; GD, Gaucher's disease; HC, healthy controls.

*, L444P (RecNcil recombinant)

[1], Generated in (Schöndorf et al., 2014)

[2], Generated using ZFN as described in Methods

[3], Generated using CRISPR-Cas9 as described in Methods

[4], Generated in (Schondorf et al., 2018)

Schöndorf, D.C., Aureli, M., McAllister, F.E., Hindley, C.J., Mayer, F., Schmid, B., Sardi, S.P., Valsecchi, M., Hoffmann, S., Schwarz, L.K., *et al.* (2014). iPSC-derived neurons from GBA1-associated Parkinson's disease patients show autophagic defects and impaired calcium homeostasis. Nat Commun *5*, 4028.

Schondorf, D.C., Ivanyuk, D., Baden, P., Sanchez-Martinez, A., De Cicco, S., Yu, C., Giunta, I., Schwarz, L.K., Di Napoli, G., Panagiotakopoulou, V., *et al.* (2018). The NAD+ Precursor Nicotinamide Riboside Rescues Mitochondrial Defects and Neuronal Loss in iPSC and Fly Models of Parkinson's Disease. Cell Rep *23*, 2976-2988.

Supplementary Table 2. Primer and sgRNA/ssODN sequences.

**Primers for stable cell line generation (pcDNA5/frt/to plasmid) (5'-3')**		
CMV	FW	CGC AAA TGG GCG GTA GGC GTG
BGH	RV	TAG AAG GCA CAG TCG AGG
**Site-directed mutagenesis (5'-3')**		
L444P	FW	GTG CCA CTG CGT CCG GGT CGT TCT TCT GA
	RV	TCA GAA GAA CGA CCC GGA CGC AGT GGC AC
E326K	FW	GGC GGT GTG TCT TCC CTA GGG TGG C
	RV	GCC ACC CTA GGG AAG ACA CAC CGC C
**Primers for genomic *GBA1* and cDNA amplification and sequencing (5'-3')**		
GBA1 Exon 8	FW	TGT GTG CAA GGT CCA GGA TCA G
	RV	TTT GCA GGA AGG GAG ACT GG
GBA1 Exon 10	FW	CAG GAG TTA TGG GGT GGG TC
	RV	GAG GCA CAT CCT TAG AGG AG
GBA1 Exon 11	RV	ACC ACC TAG AGG GGA AAG TG
GBA1 Exon 8 cDNA	FW	CGA GAC TTC ATT GCC CGT GA
	RV	TTG GGT CCT CCT TCG GGG
GBA1 Exon 10 cDNA	FW	CGT AAC TTT GTC GAC AGT CC
	RV	GCT GTT GTG GTC GTG CTA AA
**Primers for genomic *GBA1* and cDNA amplification and sequencing (5'-3')**		
GBA1 Exon 8	FW	TGT GTG CAA GGT CCA GGA TCA G
	RV	TTT GCA GGA AGG GAG ACT GG
GBA1 Exon 10	FW	CAG GAG TTA TGG GGT GGG TC
	RV	GAG GCA CAT CCT TAG AGG AG
GBA1 Exon 11	RV	ACC ACC TAG AGG GGA AAG TG
GBA1 Exon 8 cDNA	FW	CGA GAC TTC ATT GCC CGT GA
	RV	TTG GGT CCT CCT TCG GGG
GBA1 Exon 10 cDNA	FW	CGT AAC TTT GTC GAC AGT CC
	RV	GCT GTT GTG GTC GTG CTA AA
**sgRNAs and ssODN sequences**		
crRNA		CAG GCG GTG TGT CTT CCC TA
E326K ssODN GC		CAT TGC TGT ACA TTG GTA CCT GGA CTT TCT GGC TCC AGC CAA AGC CAC CCT AGG GGA GAC ACA CCG CCT GTT CCC CAA CAC CAT GCT CTT TGC CTC AGA GGC CTG TGT GGG
**Analysis of off-target effects (5'-3')**		
NUDCD3	FW	TGC CCC TTC CTC TCT AGA CC
	RV	TCA AGG CCA TCT TGT GAC CC
BBS9	FW	CCA CAC GCA CCG AGG G
	RV	AGC ACT TAC AAC GGA CGC CA
RP11-576I22.2	FW	GTT TGT GTT AGC GGA AGG CG

	RV	TGG CAA CCA GAA GTG AAT GC
WDR1	FW	ACC CCT GGA ACA AAC TGG TG
	RV	CAT TTT GTC ACG GAA CCG CA
GBAP1	FW	TCT TTG GTG AGA CTA CTA ATG GG
	RV	CAG GGT GAC TTG TTC TTC CTT TG
**qRT-PCR (5'-3')**		
ATF4	FW	GTC CCT CCA ACA ACA GCA AG
	RV	CTA TAC CCA ACA GGG CAT CC
LONP1	FW	CCC GCG CTT TAT CAA GAT T
	RV	AGA AAG ACG CCG ACA TAA GG
HSP60	FW	TGA CCC AAC AAA GGT TGT GA
	RV	CAT ACC ACC TCC CAT TCC AC
HSPA9	FW	GGA AGC TGC TGA AAA GGC TA
	RV	CTT GGG TCC AGA AGA ATC CA
DDIT3	FW	AGC CAA AAT CAG AGC TGG AA
	RV	TGG ATC AGT CTG GAA AAG CA
β-Actin	FW	TGA CCC AGA TCA TGT TTG AGA
	RV	AGT CCA TCA CGA TGC CAG T

Supplementary Table 3. List of antibodies.

TARGET	HOST	ICC	WB/*IP	Brand	Cat ID/RRID
ATP5A	Mouse		1:2000	Abcam	ab14748/ RRID:AB_301447
ATP5B	Mouse		1:1000	Santa Cruz Biotechnology	sc-135903/ RRID:AB_2061898
Calnexin (TO-5)	Mouse		1:200	Santa Cruz Biotechnology	sc-80645/ RRID:AB_1119919
DJ-1	Mouse		1:1000	Santa Cruz Biotechnology	sc-55572/ RRID:AB_831639
DOPA Decarboxylase	Rabbit	1:250		Thermo Fisher	PA5-25450/ RRID:AB_2542950
Dopamine Transporter	Rabbit	1:100		Proteintech	22524-1-AP/ RRID:AB_2879116
DYKDDDDK Tag (Flag)	Rabbit	1:1000	1:1000	Cell Signaling Technology	14793S/ RRID:AB_2572291
FOXA2	Goat	1:100		Santa Cruz Biotechnology	Sc-6553/ RRID:AB_2104865
FOXA2	Mouse	1:100		Santa Cruz Biotechnology	Sc-101060/ RRID:AB_1124660
GAPDH	Mouse		1:10000	Santa Cruz Biotechnology	sc-47724/ RRID:AB_627678
GCase	Mouse		1:1000	Sigma Aldrich	WM0002629M1/ RRID:AB_1841779
GCase	Rabbit	1:100		Proteintech	20622-1-AP/ RRID:AB_10696317
GCase MaxPab	Rabbit		*10 µg	Abnova	H00002629-D01/ RRID:AB_10658489
GFP	Mouse	1:500		Sigma Aldrich	G6539/ RRID:AB_259941
GRP78	Rabbit		1:8000	Proteintech	11587-1-AP/ RRID:AB_2119855
GRP94	Rabbit		1:1000	Proteintech	14700-1-AP/ RRID:AB_2233347
HEXB	Mouse		1:1000	Santa Cruz Biotechnology	Sc-376781/ RRID:AB_2909474
HSC70	Mouse		1:1000	Santa Cruz Biotechnology	sc-7298/ RRID:AB_627761
HSC70/HSPA8	Rabbit		1:1000	Cell Signaling Technology	8444S/ RRID:AB_10831837

HSP60	Rabbit	1:2000		Cell Signaling Technology	12165/ RRID:AB_2636980
HSP60	Mouse	1:2000	*1:3000	Santa Cruz Biotechnology	sc-271215/ RRID:AB_10607973
LAMP1	Mouse		1:100	DSHB	H4A3/RRID:AB_2296838
LIMP2	Rabbit		*1:500	ProSci	4621/RRID:AB_10904468
LIMP2	Mouse		1:1000	Santa Cruz Biotechnology	Sc-55571/ RRID:AB_831512
LONP1	Rabbit		1:2000	Proteintech	15440-1-AP/ RRID:AB_2137152
LONP1	Mouse	1:100	1:5000 *1:1000	Proteintech	66043-1-lg/ RRID:AB_2881494
MAP2	Chicken	1:2500		Biolegend	82250/ RRID:AB_291541 (previously Covance)
NDUFA10	Mouse		1:4000	Santa Cruz	sc-376357/ RRID:AB_10990305
NDUFA9	Mouse		1:5000, BNE 1:10000, SDS-PAGE	Abcam	ab14713/ RRID:AB_301431
NDUFS1	Rabbit		1:1000	GeneTex	GTX113787/ RRID:AB_2037498
NDUFS2	Mouse		1:250	Santa Cruz Biotechnology	sc-390596/ RRID:AB_2927478
OTX2	Goat	1:200		Neuromics	GT15095/ RRID:AB_2157174
S100B	Mouse	1:500		Sigma-Aldrich	S2532/ RRID:AB_477499
SDHA	Mouse		1:5000, BNE 1:10000, SDS-PAGE	Abcam	Ab14715/ RRID:AB_301433
SOX2	Mouse	1:200		Abcam	Ab75485/ RRID:AB_1278243
ß3-Tubulin	Mouse	1:1000		Biolegend	801202/ RRID:AB_10063408
ß3-Tubulin	Rabbit	1:1000		Biolegend	802001/ RRID:AB_2564645 (previously Covance)
ß-Actin	Mouse		1:5000	Santa Cruz Biotechnology	Sc-47778/ RRID:AB_2714189
Synaptophysin	Mouse	1:500		Proteintech	CL488-67864/ RRID:AB_2909472
TBC1D15	Rabbit		1:2500	Novus Biologicals	NBP2-36552/ RRID:AB_2904547

TIM23	Rabbit		1:1000	Proteintech	11123-1-AP/ RRID:AB_615045
TIM23	Mouse		1:1000	BD bioscience	611222 / RRID:AB_398754
TIMMDC1	Rabbit		1:1000	Atlas Antibodies - Sigma	HPA053214, RRID:AB_2682081
TIMMDC1 (F-1)	Mouse		1:100	Santa Cruz Biotechnology	Sc-514926 /AB_2924833
TIMMDC1 (G-10)	Mouse		1:1000 *1:100	Santa Cruz Biotechnology	sc-514927/ RRID:AB_2909473
TOM20	Mouse	1:500	1:1000	Santa Cruz Biotechnology	sc-11415/ RRID:AB_2207533
TOM70	Rabbit		1:1000	ProteinTech	14528-1-AP/ RRID:AB_2303727
Tyrosine Hydroxylase	Rabbit	1:500	1:1000	Pel-Freez Biologicals	P40101-150/ RRID:AB_2617184
Vimentin	Mouse		1:2000	BD Biosciences	550513, RRID:AB_393716
Vinculin	Mouse		1:1000	Santa Cruz Biotechnology	sc-73614/ RRID:AB_1131294
α-Synuclein	Rabbit		1:1000	Millipore	04-1053, RRID:AB_1977522
α-Synuclein	Mouse	1:250	1:1000	BD Bioscience	610787/ RRID:AB_398108
α-Synuclein Phospho (Ser129)	Mouse	1:500		Biolegend	825701/ RRID:AB_2564891
Normal Rabbit IgG	Rabbit		*10 µg	Covalab	pab01004-P, RRID: AB_2934307

Secondary antibodies

Anti-Rabbit IgG (H+L) Alexa Fluor™ 488	Goat	1:1000		Invitrogen	A-11008 RRID:AB_143165
Anti-Rabbit IgG (H+L) Alexa Fluor™ 568	Goat	1:1000		Invitrogen	A-11011 RRID:AB_143157
Anti-Mouse IgG (H+L) Alexa Fluor™ 488	Goat	1:1000		Invitrogen	A-11001 RRID:AB_2534069
Anti-Mouse IgG (H+L) Alexa Fluor™ 568	Goat	1:1000		Invitrogen	A-11004 RRID:AB_2534072
Anti-Chicken IgG (H+L) Alexa Fluor™ 647	Goat	1:1000		Invitrogen	A-21449 RRID:AB_2535866

Anti-Rat IgG (H+L) Alexa Fluor™ 568	Goat	1:1000		Abcam	Ab175476 RRID:AB_2813739
Anti-Goat IgG (H+L) Alexa Fluor™ 488	Donkey	1:1000		Invitrogen	A-11055 RRID:AB_2534102
Anti-Rabbit IgG (H+L) Alexa Fluor™ 568	Donkey	1:1000		Invitrogen	A-10042 RRID:AB_2534017
Anti-Sheep IgG (H+L) Alexa Fluor™ 488	Donkey	1:1000		Invitrogen	A-11015 RRID:AB_141362
Anti-mouse IgG, HRP-linked Antibody	Horse		1:5000	Cell Signaling Technology	Cat# 7076, RRID:AB_330924
Anti-rabbit IgG, HRP-linked Antibody	Goat		1:5000	Cell Signaling Technology	Cat# 7074, RRID:AB_2099233
IRDye® 680RD Goat anti-Mouse IgG	Goat		1:10000	LI-COR Biosciences	926-68070, RRID:AB_10956588
IRDye 800CW Goat anti-Rabbit IgG	Goat		1:10000	LI-COR Biosciences	926-32211, RRID:AB_621843

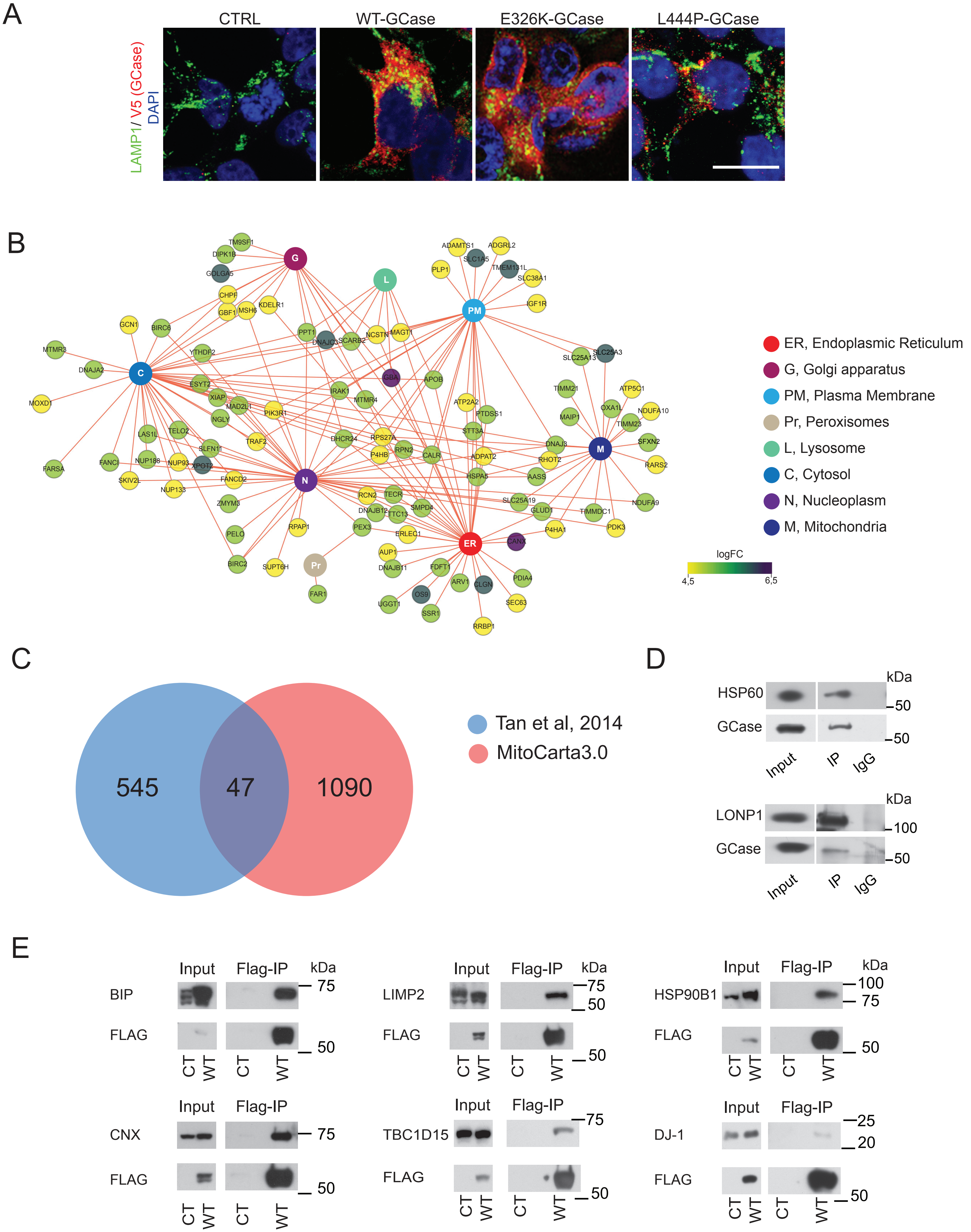

Supplementary Figure 1

Supplementary Figure 1. Characterization and validation of T-Rex HEK cells.

(A) Representative confocal images showing LAMP1 (green) and GCase (red) in WT and mutant (E326K and L444P)-GCase T-Rex HEK cells. Scale bar, 20 µm. **(B)** Representation of the top 100 WT-GCase interactors and their subcellular localization generated with Cytoscape version 3.9.0. **(C)** Venn diagram showing the overlap between the endogenous WT-GCase interactome (Tan et al., 2014) and human mitochondrial proteins according to the Human Micarta 3.0 database**.** **(D)** HSP60 and LONP1 immunoprecipitation showing the interaction of HSP60 and LONP1 with V5-Flag-tagged WT-GCase. **(E)** CoIP validation of GCase interactors with known localization in the ER (BIP and calnexin), cytosol (LIMP2, TBC1D15, and HSP90B1), and mitochondria (DJ-1). T-Rex HEK cells expressing V5-Flag only were used as a control. Western blot analysis of 20 µg of the whole-cell lysates prior to immunoprecipitation was used to visualize protein input.

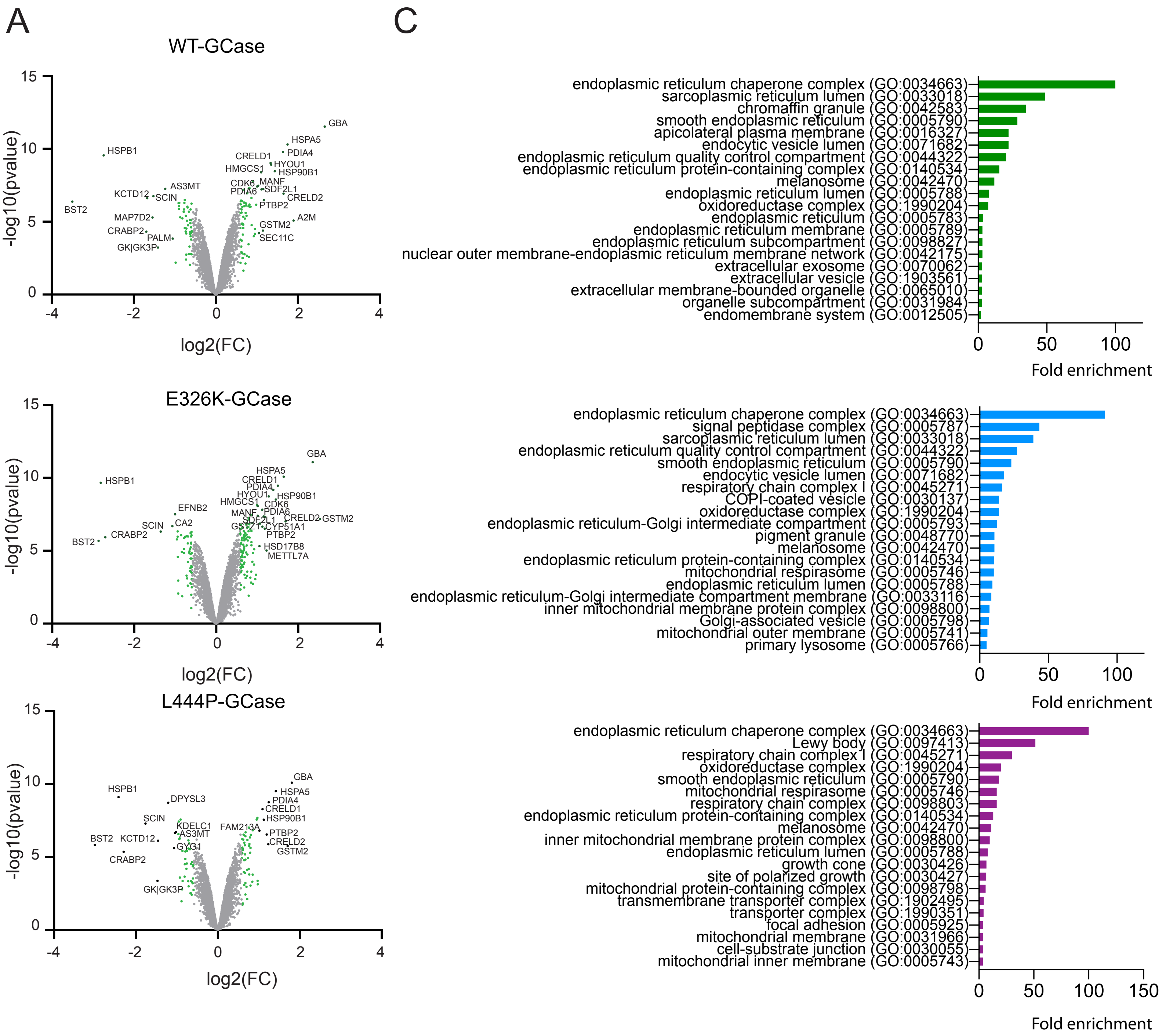

B

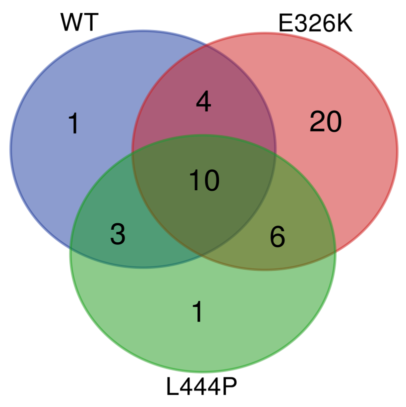

WT GCase CARS, IDI1, DNAJC3, NAXD, DCXR, DLD, GPX4, ACO2, FAM213A, HDHD5, BAD, NDUFA7, NDUFA12, NDUFS4, VDAC3, TOMM5, TOMM40, GK3P

E326K GCase IDI1, DLD, WARS, GPX4, ISCA2, NSUN2, EXD2, OXCT1, ACO2, NAXD, DNAJC3, DHRS7, FIS1, ACADS, SDHAF3, BCL2L13, HDHD5, ARG2, ARMC10, DCXR, ACOT2, ALKBH1, ALDH6A1, NDUFS5, MTX3, NDUFC2, BPHL, NDUFA10, UQCRB, NDUFA7, GDAP1, NDUFS4, NDUFA12, PUSL1, TOMM5, VDAC3, MARC1, GK3P, GSTZ1, HSD17B8

L444P GCase OXCT1, CARS, GPX4, NAXD, DLD, FIS1, HDHD5, DCXR, HSD17B8, NDUFS5, NDUFC2, NDUFA7, NDUFA10, NDUFA12, NDUFB8, NDUFS4, BAD, TOMM5, GK3P, FAM213A

Supplementary Figure 2

Supplementary Figure 2. Overexpression of mutant GCase leads to the dysregulation of mitochondrial pathways. (A) TMT whole-cell lysate proteome of T-Rex HEK cells expressing WT or mutant GCase (E326K or L444P). Volcano plot showing DEPs in WT-, E326K-, and L444P-GCase lines compared to the control. Hit interactors that differ by 2-fold and have a false discovery rate (FDR) of ≤5% are labeled. Limma-based differential analysis was used. **(B)** Venn diagram showing the differentially expressed mitochondrial proteins in WT or mutant GCase (E326K or L444P) compared to the control. **(C)** Top enriched biological processes (BPs) of DEPs in WT or mutant GCase (E326K or L444P) compared to the control.

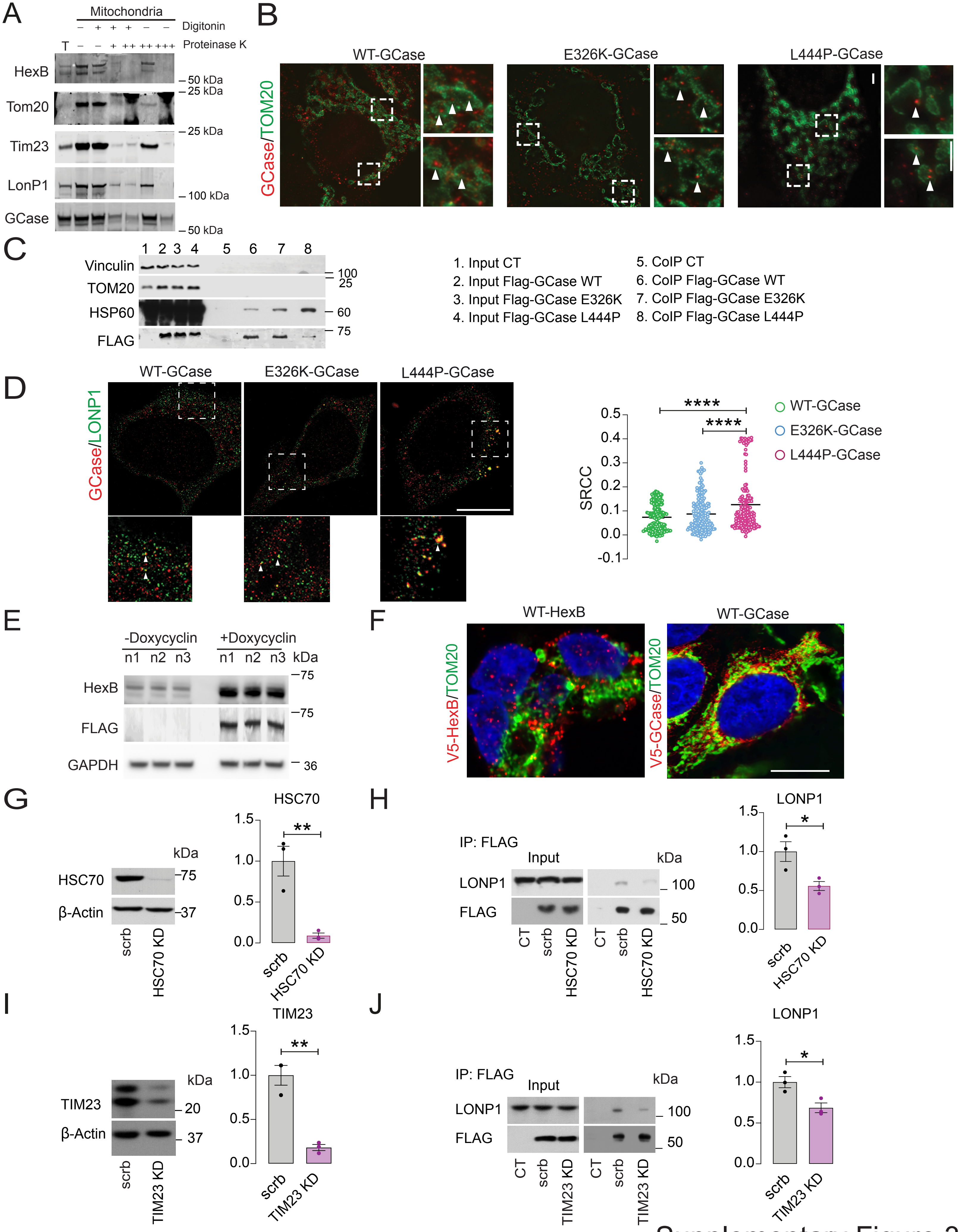

Supplementary Figure 3

Supplementary Figure 3. GCase is imported into mitochondria by the mitochondrial protein import machinery and interacts with mitochondrial chaperones and proteases in T-Rex HEK cells. (A) Isolated mitochondria from WT-GCase T-Rex HEK cells were solubilized with 20 ng/μl digitonin and subjected to digestion with proteinase K (PK), and immunoblotting was performed for the indicated markers. PK concentration: +, 0,2 mg/mL; ++, 2 mg/mL; +++, 6 mg/mL. **(B)** ExM images showing TOM20 (green) and GCase (red) in WT and mutant (E326K and L444P)-GCase T-Rex HEK cells. Scale bar, 1 µm. **(C)** Validation of the FLAG-IP using the non-interacting proteins TOM20 and Vinculin, and the mitochondrial interactor HSP60**. (D)** ExM images after LONP1 (green) and GCase (red) staining in WT and mutant T-Rex HEK cells. Colocalization analysis of LONP1/GCase in T-Rex HEK cells. Mean ± SEM; one-way ANOVA with Bonferroni post hoc test, **** $P<0.001$. Five images taken from $n = 3$ independent experiments. Scale bar, 10 µm. **(E)** Representative western blot of FLAG and HEXB in uninduced and induced T-Rex HEK cells overexpressing HEXB. **(F)** Representative confocal images showing HEXB and GCase intracellular localization in HEXB and GCase T-Rex cells. HEXB/V5 (red), GCase (red), TOM20 (green). Scale bar, 10 µm. **(G)** Representative western blot analysis and validation of the knockdown (KD) efficiency of HSC70 shRNA at the protein level in L444P-GCase T-Rex HEK cells. Mean ± SEM; unpaired two-tailed t test, ** $P=0.0079$; $n=3$ independent experiments. **(H)** Validation of GCase interaction with LONP1 after HSC70 KD by coimmunoprecipitation followed by western blot analysis and densitometry quantification in L444P-GCase T-Rex HEK cells. Mean ± SEM; unpaired two-tailed t test, * $P=0.0342$; $n=3$ independent experiments. **(I)** Representative western blot analysis and validation of the KD efficiency of TIM23 shRNA at the protein level in WT-GCase T-Rex HEK cells. Mean ± SEM; unpaired two-tailed t test, ** $P=0.0022$; $n=3$ independent experiments. **(J)** Validation of GCase interaction with LONP1 after TIM23 KD by coimmunoprecipitation followed by western blot analysis and densitometry quantification in L444P-GCase T-Rex HEK cells. Mean ± SEM; unpaired two-tailed t test, * $P=0.0255$; $n=3$ independent experiments.

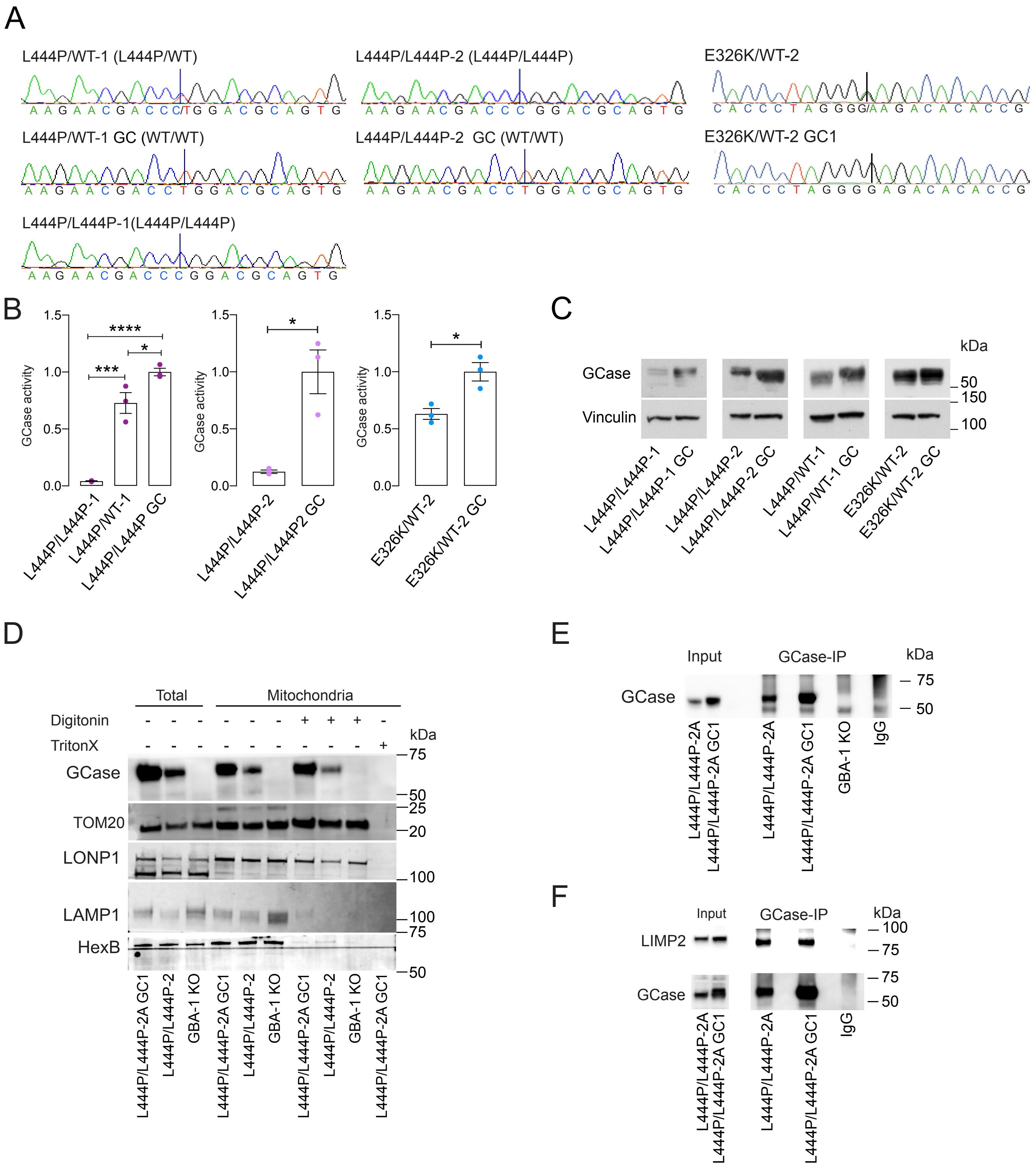

Supplementary Figure 4

Supplementary Figure 4. Generation and characterization of isogenic human *GBA1* mutant iPSC lines. (A) Sanger sequencing of *GBA1* mutant and gene-corrected (GC) isogenic control iPSCs. The line indicates the mutation site. **(B)** Activity of GCase in *GBA1* mutant and isogenic control iPSC-derived neurons (relative to isogenic controls). Mean ± SEM; unpaired two-tailed t test or one-way ANOVA with Bonferroni post hoc test; left panel: **** P<0.0001, *** P=0.0004, * P=0.0413; middle panel: * P=0.0103; right panel: * P=0.0167; n=3 independent experiments. **(C)** Representative western blots showing GCase levels in *GBA1* PD and isogenic control iPSC-derived neurons. **(D)** Isolated mitochondria from the L444P/L444P, isogenic control, and *GBA1* KO NPCs were solubilized with digitonin and Triton X-100 (solubilization of mitochondrial inner and outer membranes). Immunoblotting was performed for the indicated markers. **(E)** Immunoprecipitation of endogenous GCase from human L444P/L444P, isogenic control, and *GBA1* KO NPCs followed by western blot analysis demonstrating the efficiency and specificity of GCase pull-down. **(F)** Immunoprecipitation of endogenous GCase followed by western blot analysis demonstrating GCase interaction with LIMP2.

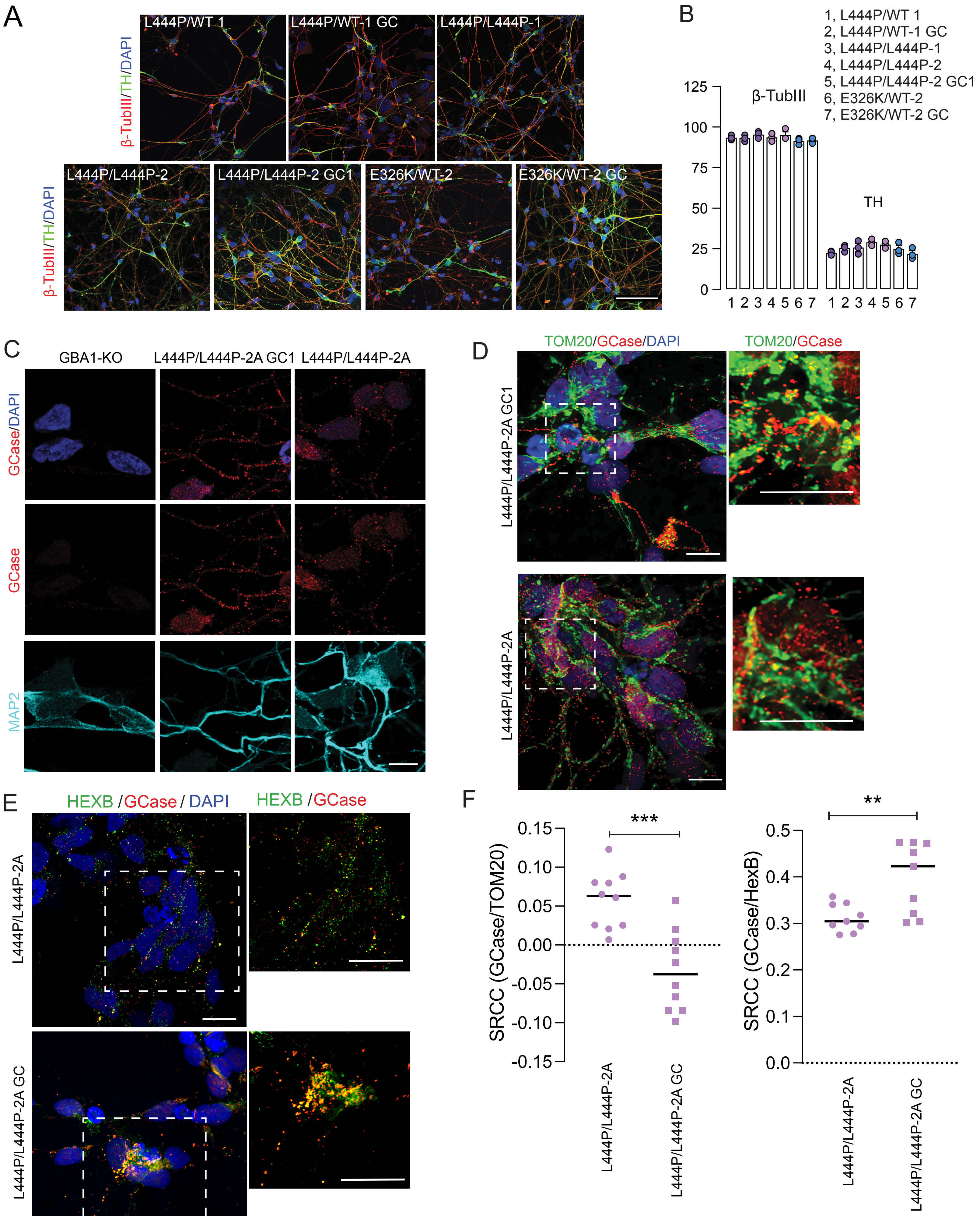

Supplementary Figure 5

Supplementary Figure 5. Characterization of human iPSC-derived neurons and localization of GCase.

(A) *GBA1* PD and isogenic control iPSCs were differentiated into DA neurons. Immunostaining of the indicated differentiated iPSC cultures at 35 DIV. Cells were stained for β-III-tubulin (β-TUBIII, red) and TH (green). Nuclei were counterstained with DAPI (blue). Representative images of the indicated iPSC lines are shown. Scale bar, 50 μm. **(B)** Quantification of β-TUBIII/DAPI- and TH/DAPI-positive cells in differentiated iPSC cultures at 35 DIV. Mean ± SEM; n = 3 independent differentiations. **(C-E)** Immunostaining of the indicated differentiated iPSC cultures. **(C)** Cells were stained for GCase (red) and MAP2 (cyan). Scale bar, 10 μm. **(D)** Cells were stained for GCase (red) and TOM20 (green). Scale bar, 10 μm. **(E)** Cells were stained for GCase (red) and HEXB (green). Nuclei were counterstained with DAPI (blue). Scale bar, 10 μm. **(F)** Colocalization analysis of GCase/TOM20 and GCase/HEXB. Mean ± SEM; unpaired two-tailed t test, *** P=0.0003, ** P=0.0064; GCase/TOM20 (n=10 cells), GCase/HEXB (n=9 cells), from 3 independent batches.

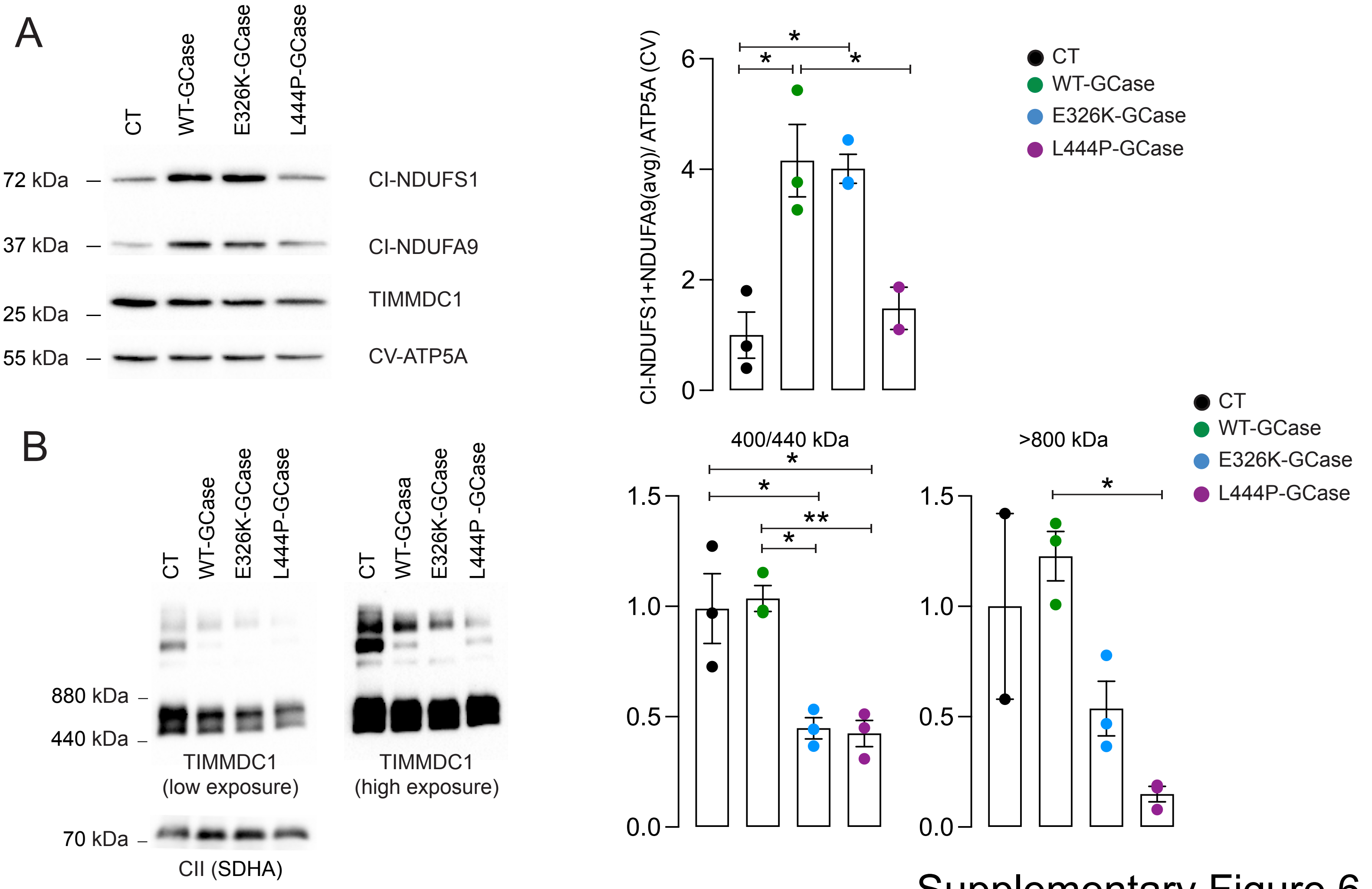

Supplementary Figure 6

Supplementary Figure 6. Analysis of mitochondrial complexes in T-Rex HEK cells. (A) Western blot analysis of NDUFS1, NDUFA9, TIMMDC1, and ATP5A in T-Rex HEK cells overexpressing V5-Flag-GCase (WT, L444P, or E326K). Densitometric quantification of the average of CI subunits signal normalized to CV-ATP5A. Mean ± SEM; one-way ANOVA with Bonferroni post hoc test; * P=0.0103, 0.0133, 0.0443 in sequence; n = 3 independent experiments. (**B**) BN-PAGE, followed by immunotransfer to a nitrocellulose membrane, was probed with an antibody against TIMMDC1. Quantification of TIMMDC1 complexes at ~400/440 kDa and the higher-molecular–weight species of >800 kDa is shown on the right. Mean ± SEM; one-way ANOVA with Bonferroni post hoc test; left panel: ** P=0.0097, * P=0.0196, 0.0153, 0.0123 in sequence; right panel * P=0.0117, n = 3 independent experiments.

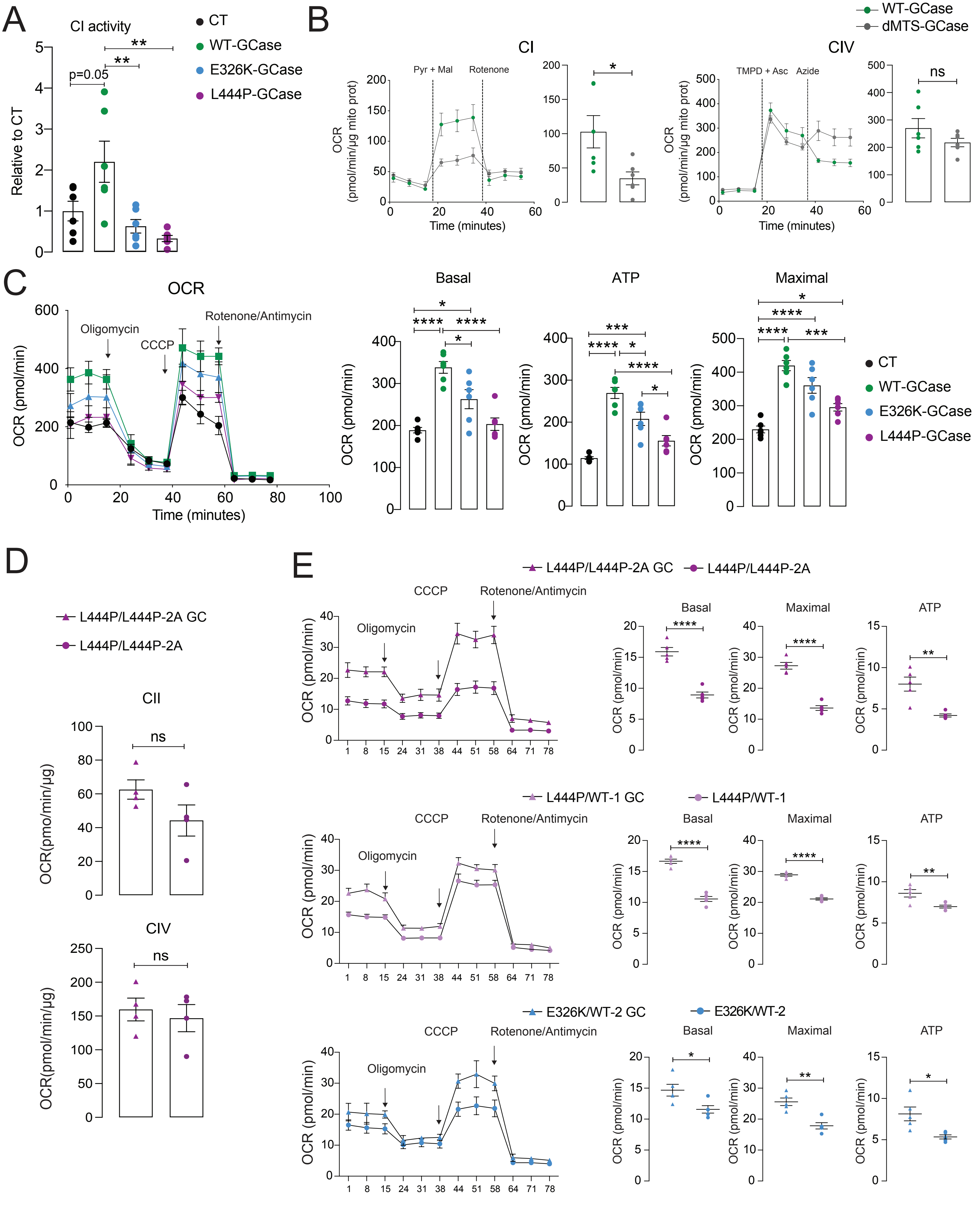

Supplementary Figure 7

Supplementary Figure 7. *GBA1* mutations alter CI activity and mitochondrial respiration in stable T-Rex HEK cells and iPSC-derived neurons. (A) Enzymatic assays for complex I (CI) activity in mitochondrial extracts from WT-, E326K-, and L444P-GCase T-Rex HEK cells. Mean ± SEM; one-way ANOVA with Bonferroni post hoc test, ** P=0.0067, 0.0012 in sequence; n=6 independent experiments. **(B)** CI and complex IV (CIV) activity in mitochondrial extracts from WT- and dMTS-GCase T-Rex HEK cells. Mean ± SEM, unpaired two-tailed t test, * P=0.0235; n=6 independent experiments. **(C)** Mitochondrial respiration in WT-, E326K-, and L444P-GCase T-Rex HEK cells. Oxygen consumption rates (OCR) were measured over time after the sequential addition of oligomycin, CCCP and rotenone/antimycin A. Values for the basal OCR, ATP-linked OCR, and maximal OCR are shown. Mean ± SEM; one-way ANOVA with Bonferroni post hoc test; left panel: **** P<0.0001, * P=0.0187, 0.0160 in sequence; middle panel: **** P<0.0001, *** P=0.0001, * P= 0.0104, 0.0385 in sequence; right panel: **** P<0.0001, *** P=0.0001, * P= 0.0473; n=6 independent experiments. **(D)** CII and CIV activity in iPSC-derived neurons. Mean ± SEM; unpaired two-tailed t test, n=4 independent experiments. **(E)** Mitochondrial respiration in E326K-, L444P-, and recombinant L444P GBA1-derived iPSC neurons. The OCR was measured over time after the sequential addition of oligomycin, CCCP and rotenone/antimycin A. Values for the basal OCR, ATP-linked OCR, and maximal OCR are shown. Mean ± SEM; unpaired two-tailed t test; upper panel: **** P<0.0001, ** P=0.0024; middle panel: **** P<0.0001, ** P= 0.0099; lower panel: * P=0.0241, 0.0129, in sequence; ** P=0.0012; n=5 independent experiments.

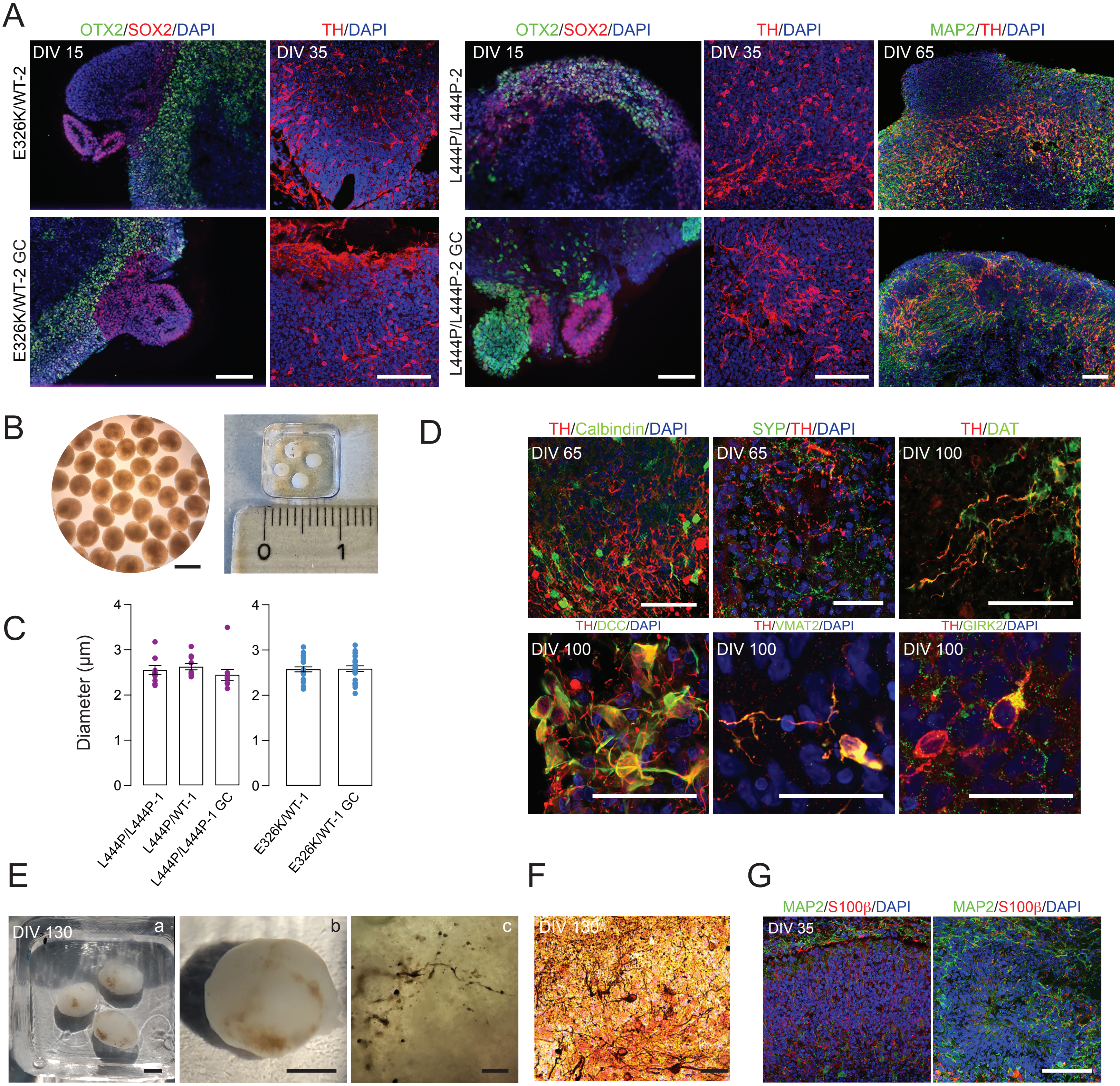

Supplementary Figure 8

Supplementary Figure 8. Generation and characterization of *GBA1* mutant midbrain organoids. (A) Immunostaining for OTX2 (green)/SOX2 (red) and MAP2 (green)/TH (red) in midbrain organoids. The genotype and DIV are indicated. Cell nuclei were counterstained with DAPI (blue). Scale bar, 100 µm. **(B)** Representative brightfield images showing EBs (left) and midbrain organoids at 65 DIV (right). Scale bar, 150 µm. **(C)** The major diameter of mutant and control midbrain organoids was measured at 65 DIV. Mean ± SEM; n=10 organoids (L444P lines) and n=22 organoids (E326K lines) from 3 different batches were measured. **(D)** Immunostaining for calbindin, synaptophysin (SYP), dopamine transporter (DAT), dopa decarboxylase (DCC), vesicular monoamine transporter 2 (VMAT2), G-protein-regulated inward-rectifier potassium channel 2 (GIRK2) (all green) and TH (red) in midbrain organoids at the indicated time points. Cell nuclei were counterstained with DAPI (blue). Scale bar, 25 µm. **(E)** Representative brightfield image of long-term cultured midbrain organoids at 130 DIV. Scale bar, 1 mm (a,b), 50 µm (c). **(F)** Fontana Masson-stained cryosection from a midbrain organoid at 130 DIV. DA neurons were visualized by TH staining with DAB chromogen. Scale bar, 20 µm. **(G)** Immunostaining for MAP2 (green) and S100B (red) in midbrain organoids at the indicated timepoints. Cell nuclei were counterstained with DAPI (blue). Scale bar, 200 µm.

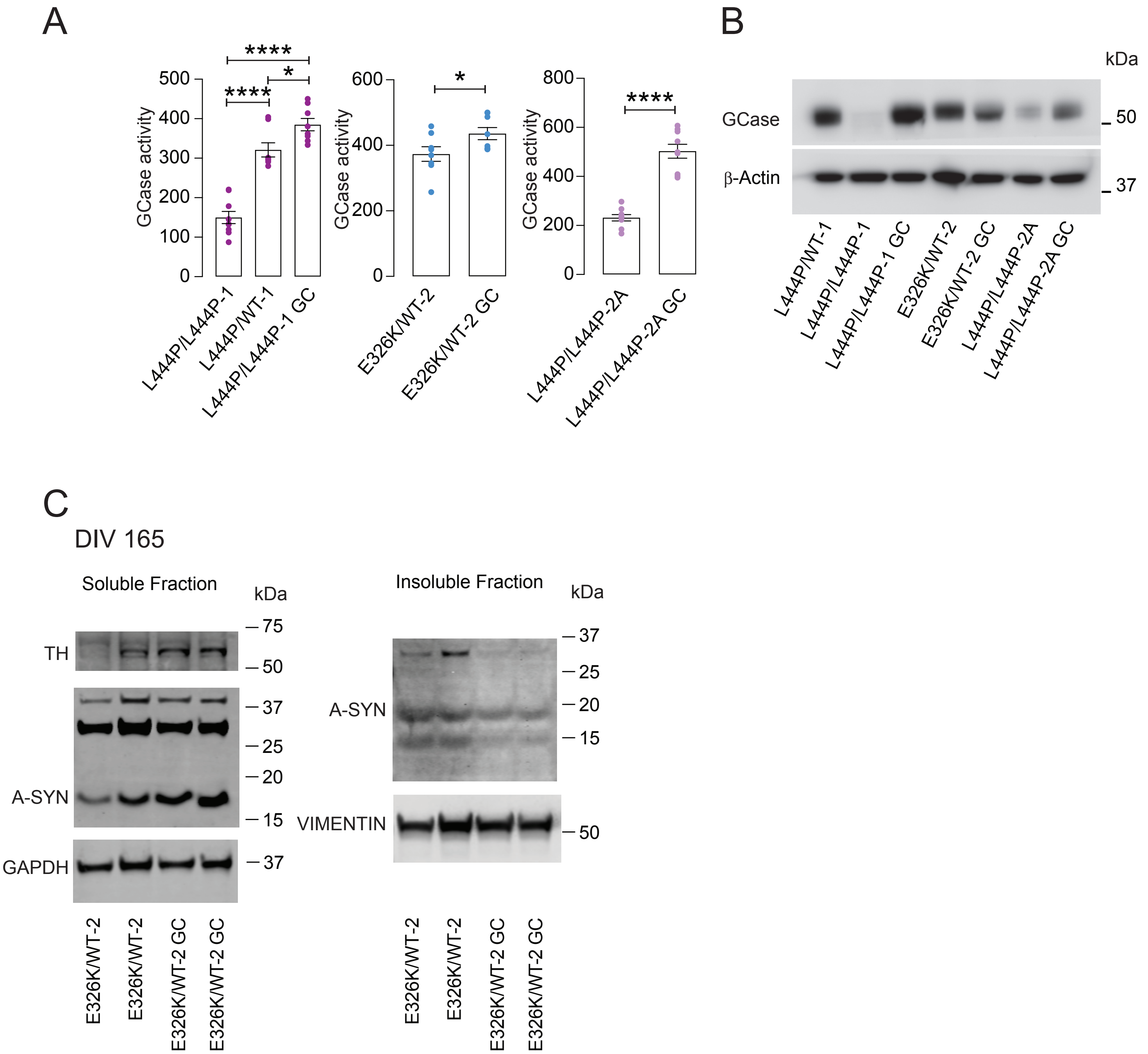

Supplementary Figure 9

Supplementary Figure 9. Analysis of GCase activity and A-SYN pathology in *GBA1* mutant midbrain organoids. **(A)** GCase activity in *GBA1* mutant organoids and corresponding isogenic controls at 42 DIV. Mean ± SEM; unpaired two-tailed t test or one-way ANOVA with Bonferroni post hoc test; left panel: **** $p < 0.0001$, * $P = 0.0384$, middle panel: * $P = 0.0474$; right panel: **** $P < 0.0001$; each dot represents an individual midbrain organoid, 3 independent differentiation onsets. **(B)** GCase protein levels in *GBA1* mutant organoids and corresponding isogenic controls at 42 DIV. **(C)** Western blots of detergent-soluble TH, A-SYN, and GAPDH, and detergent-insoluble A-SYN and Vimentin proteins in mutant and isogenic gene-corrected midbrain organoids.

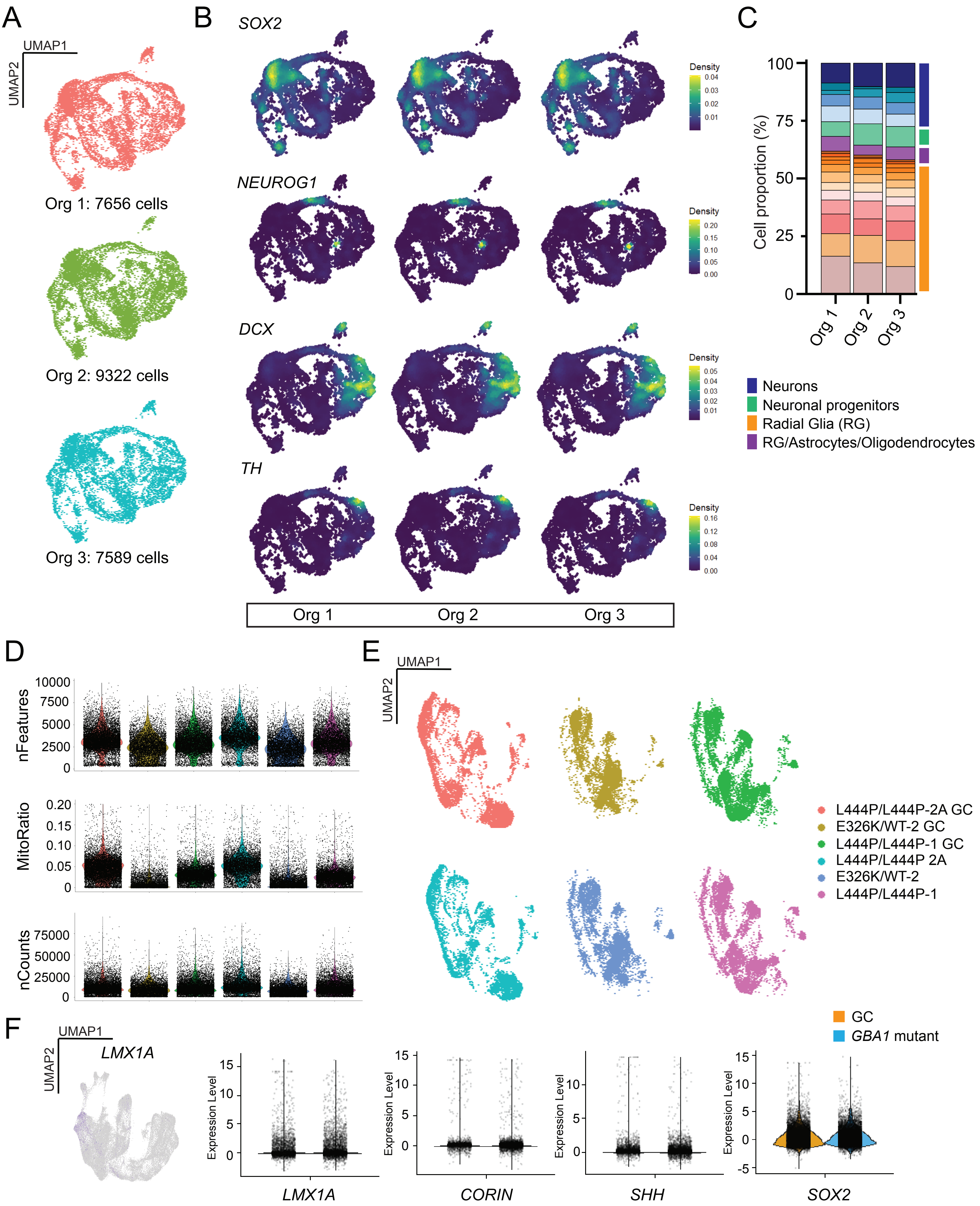

Supplementary Figure 10

Supplementary Figure 10. scRNA-seq analysis of *GBA1* mutant midbrain organoids. (A) UMAP dimensionality reduction plot from non-integrated 42 days individual midbrain organoids. Final cell number considered for the analysis is indicated in the figure. **(B)** Cells expressing *SOX2*, *NEUROG1*, *DCX*, and *TH* are plotted for each individual organoid using Nebulosa's kernel function utilizing UMAP cellular density features. **(C)** Representation of the frequency of major cell clusters in individual organoids. Cell clusters were determined by the expression of specific markers. **(D)** Violin plot illustrating the distribution of the number of expressed genes, UMI, and mitochondrial percentages in 42 days *GBA1* mutant midbrain organoids and isogenic controls. **(E)** UMAP dimensionality reduction plot showing non-integrated datasets. **(F)** UMAP and Violin plots showing floor plate markers in integrated *GBA1* mutant midbrain organoids and isogenic gene-corrected controls (GC). (L444P/L444P-2 mutant and gene-corrected lines, 8037 cells and 8198 cells, respectively; E326K/WT-1 mutant and gene-corrected lines, 6691 cells and 6223 cells, respectively; L444P/L444P-1 mutant and gene-corrected lines, 6750 cells and 9886 cells, respectively).

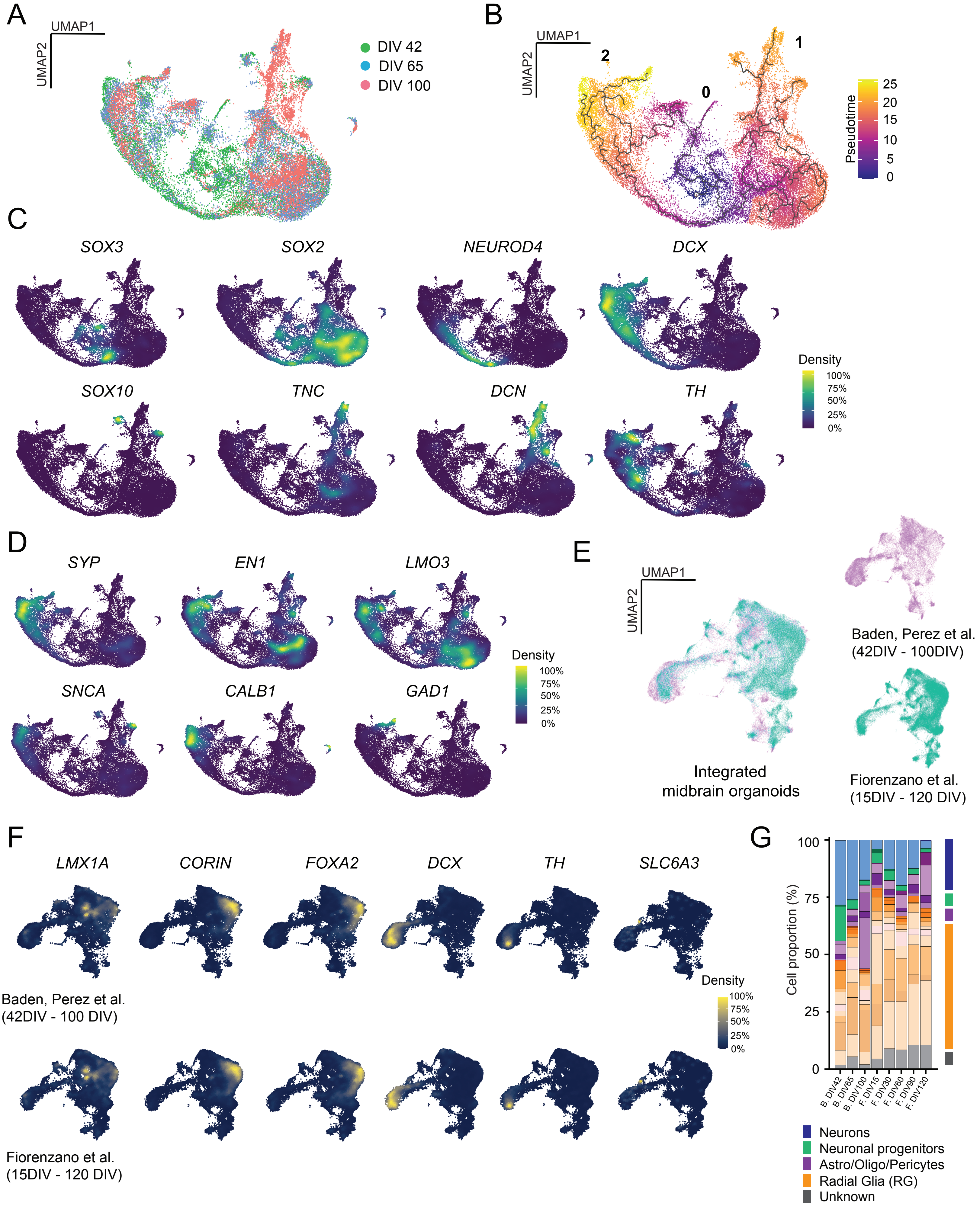

Supplementary Figure 11

Supplementary Figure 11. iPSC-derived midbrain organoids show a precise developmental cell fate trajectory similar to that of the developing human midbrain. (A) UMAP plot from integrating datasets on 42, 65, and 100-day midbrain organoids. Colors show cells sampled from different time points. **(B)** Single-cell trajectories by Monocle analysis showing the development of midbrain organoids. *SOX3*-expressing cells (State 0) were specified as roots, and the distance from a cell to the root corresponded to the pseudotime. **(C)** Nebulosa's kernel function and UMAP visualization of gene expression in emerging cellular clusters on cellular trajectories and **(D)** specific neuronal clusters. **(E)** UMAP plot from midbrain organoid integrated datasets at the indicated timepoints. **(F)** Visualization of the floor plate (*LMX1A*, *CORIN*, *FOXA2*), neuronal (*DCX*) and dopaminergic markers (*TH*, *SLC6A3*) along the UMAP visualization plot indicate similarities in cell-type distribution. **(G)** Representation of the frequency of cell clusters in pooled datasets at the indicated timepoint.

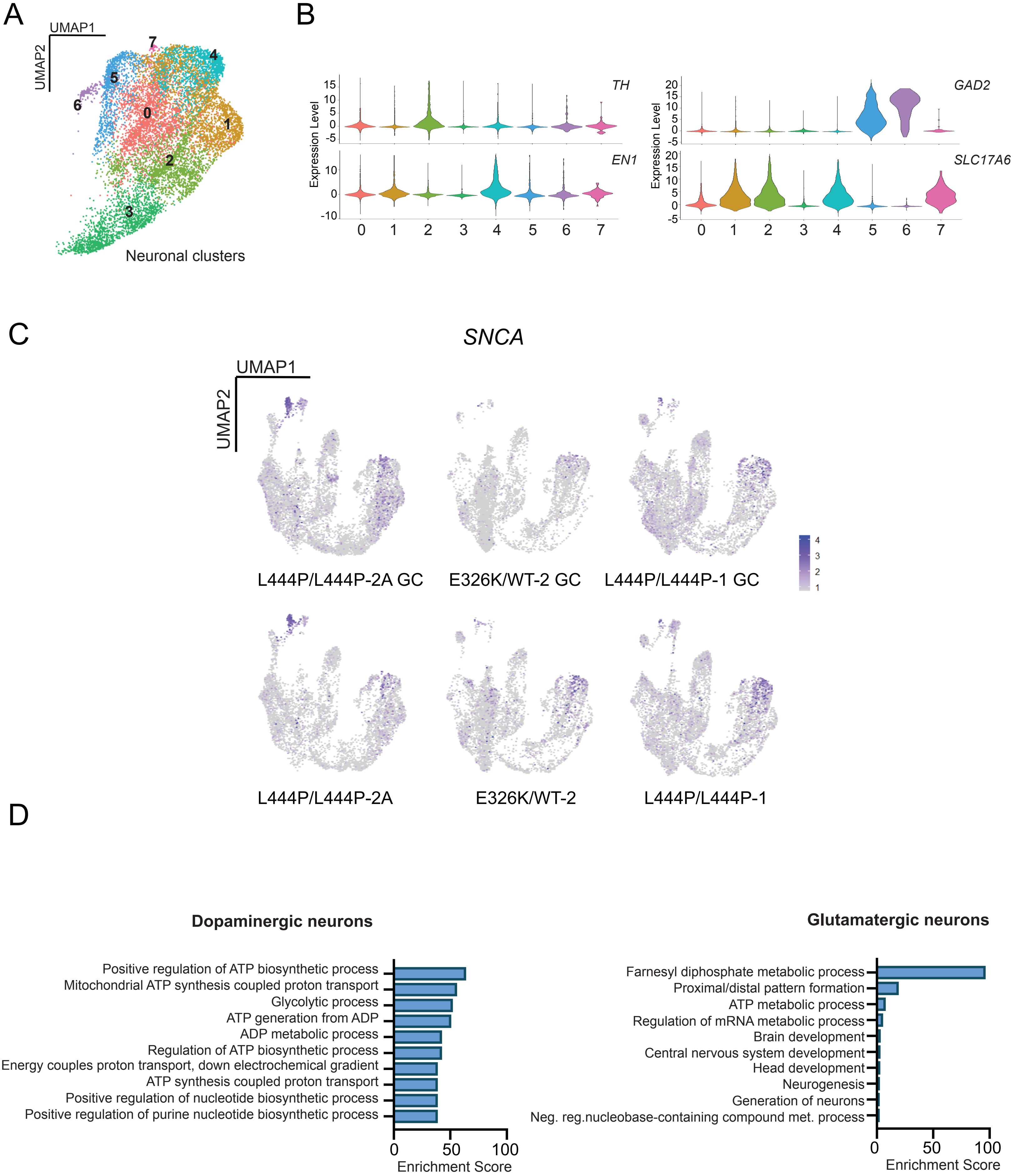

Supplementary Figure 12

Supplementary Figure 12. scRNA-seq analysis of neuronal subclusters in *GBA1* mutant midbrain organoids. (A) UMAP dimensionality reduction plot showing the neuronal subclusters. **(B)** Violin plots showing the expression levels of the indicated genes in neuronal subclusters. **(C)** UMAP dimensionality reduction plot showing *SNCA* levels in individual organoids. **(D)** Gene ontology pathway analysis showing the top enriched biological processes (BPs) in the DA and GABAergic neuronal clusters in recombinant L444P mutant midbrain organoids.

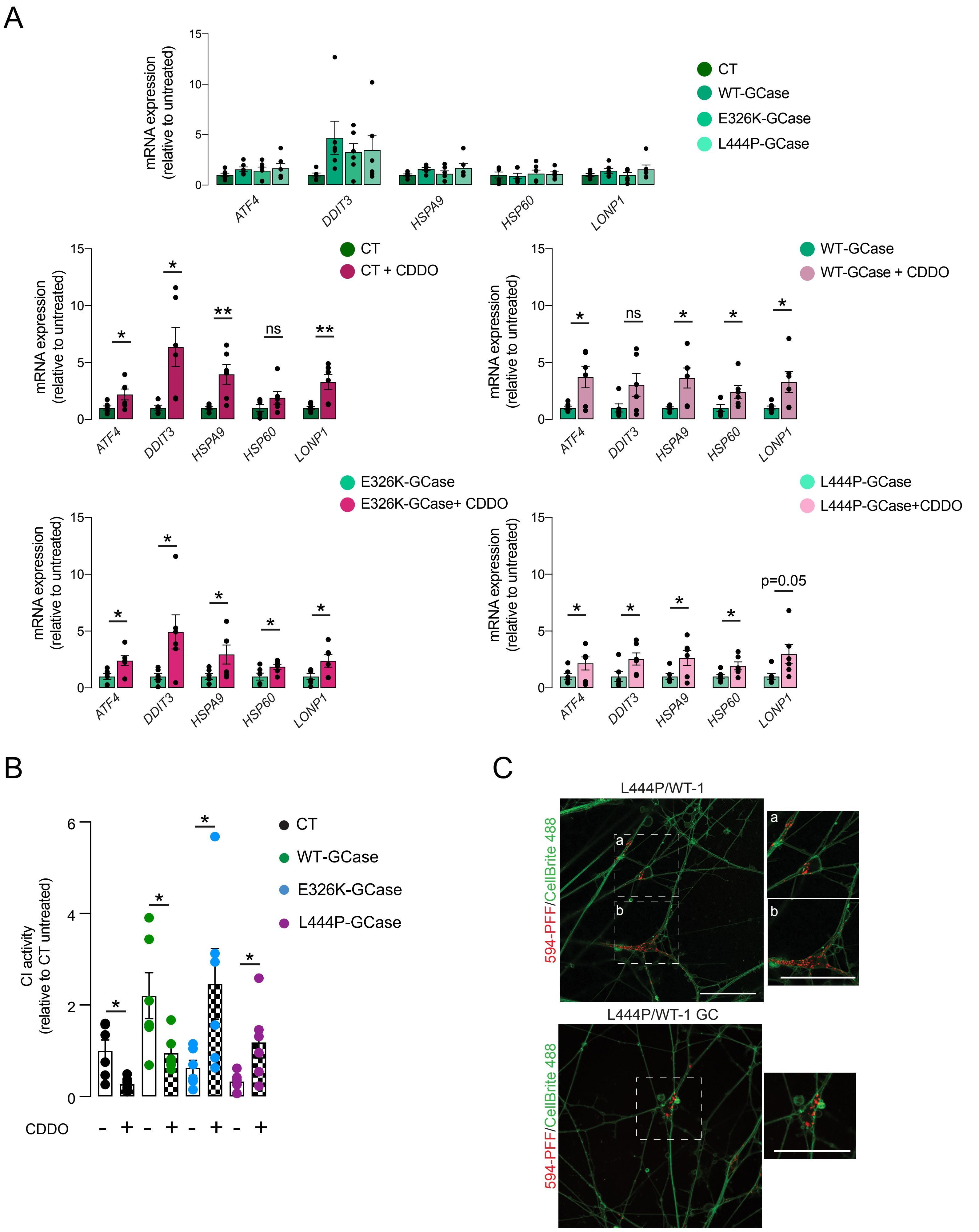

Supplementary Figure 13

Supplementary Figure 13. Inhibition of LONP1 in HEK cells and iPSC-derived neurons. (A) T-Rex HEK cells expressing WT or mutant GCase (E326K or L444P) were treated with 1 μM CDDO or left untreated, and the expression of genes involved in the mitochondrial quality system (*ATF4*, *DDIT3*, *HSPA9*, *HSP60*, *LONP1*) was analyzed with qRT–PCR. Mean ± SEM; two-tailed unpaired t test; middle panel (CT): ** P=0.0065,0.0065, * P=0.0453, 0.0109 in sequence; middle panel (WT): * P=0.0168, 0.0847, 0.0139, 0.0485, 0.036 in sequence; lower panel (E326K): * P=0.0167, 0.0275, 0.0499, 0.0480, 0.0451 in sequence; lower panel (L444P): * P= 0.0452, 0.0463, 0.0438, 0.0394 in sequence; n = 6 biological replicates. **(B)** CI activity measured in mitochondrial extracts from T-Rex HEK cells expressing WT or mutant GCase (E326K or L444P) treated with 1 μM CDDO or left untreated. Mean + SEM; two-tailed unpaired t test; * P=0.0139, 0.0393, 0.0423, 0.0341 in sequence, n = 6 biological replicates. **(C)** iPSC-derived neurons of the indicated genotypes were incubated with Alexa Fluor 594-labeled A-SYN preformed fibrils (PFFs), and live-cell imaging was performed 24 h after treatment. To analyze A-SYN localization, cell membranes were labeled with CellBrite™ 488 membrane dye, which is retained on the cell surface. Scale bar = 20 μm

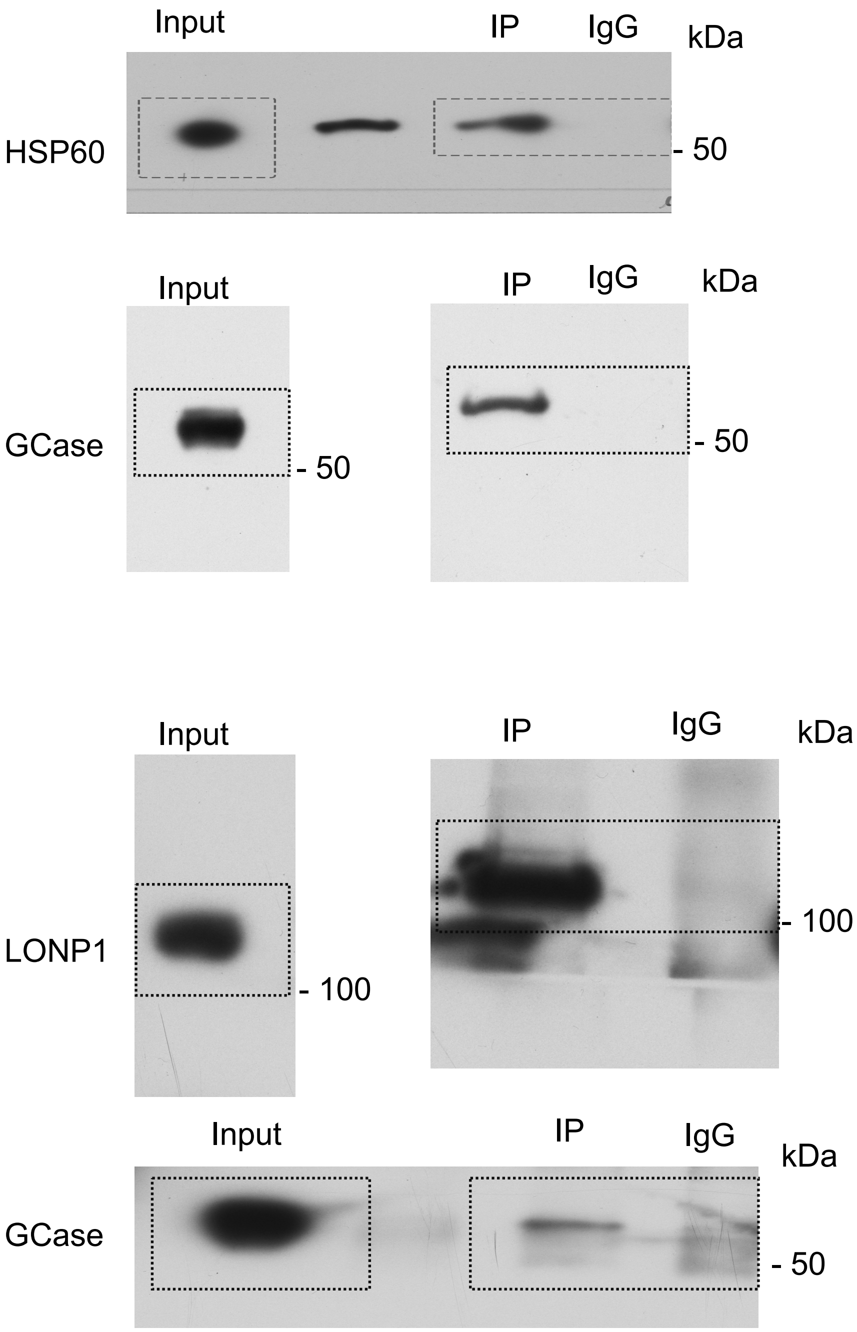

Supplementary Figure 1D

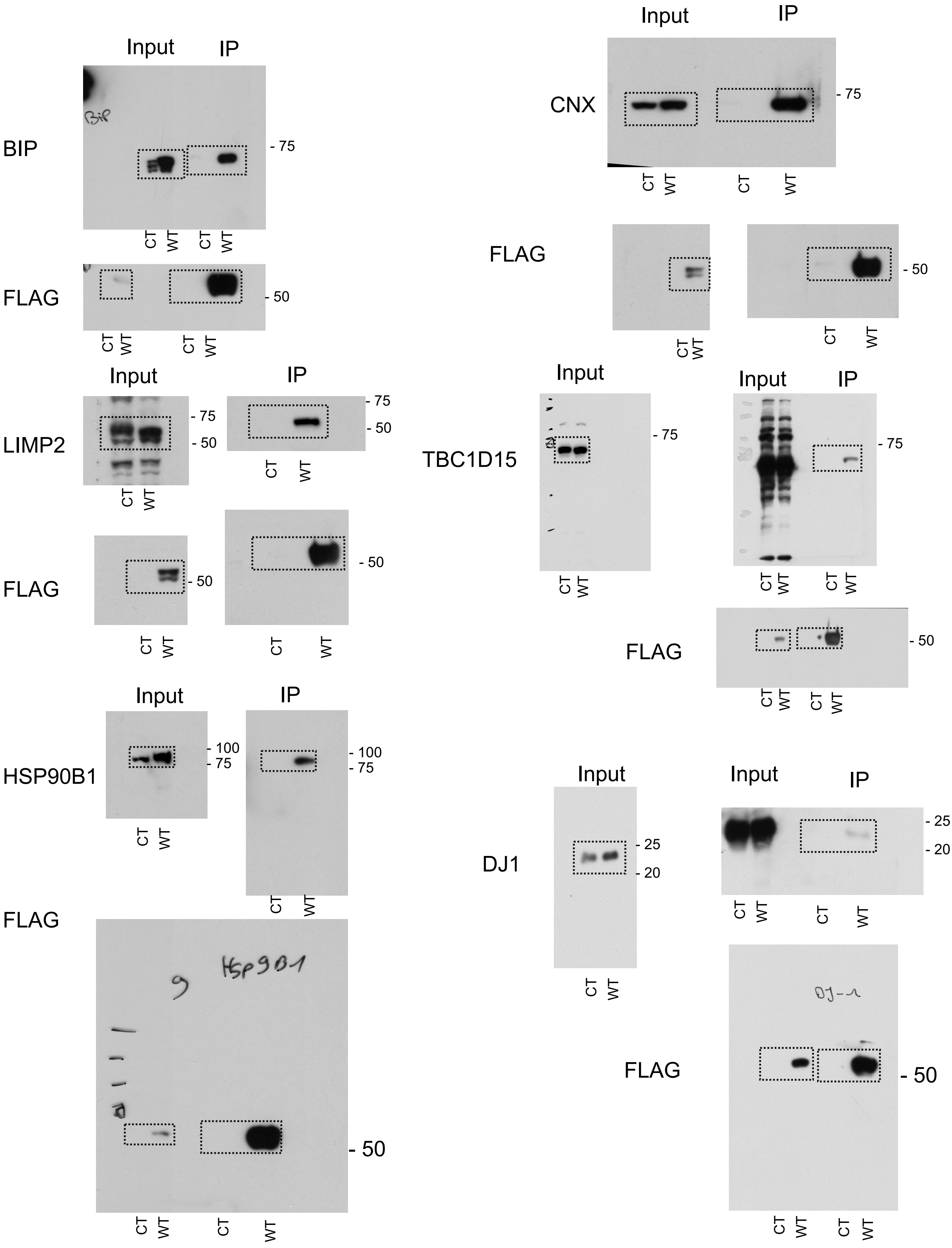

Supplementary Figure 1E

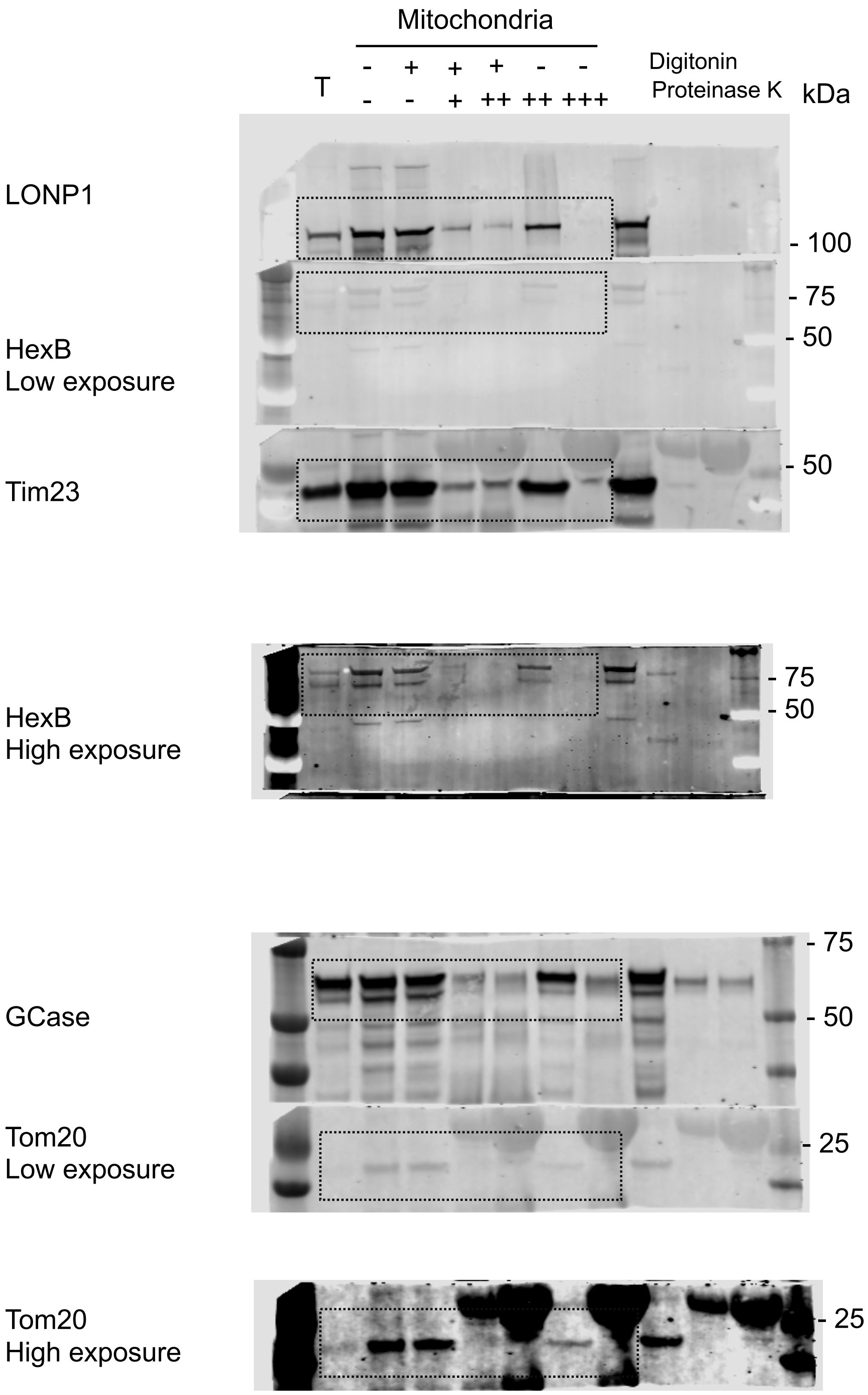

Supplementary Figure 3A

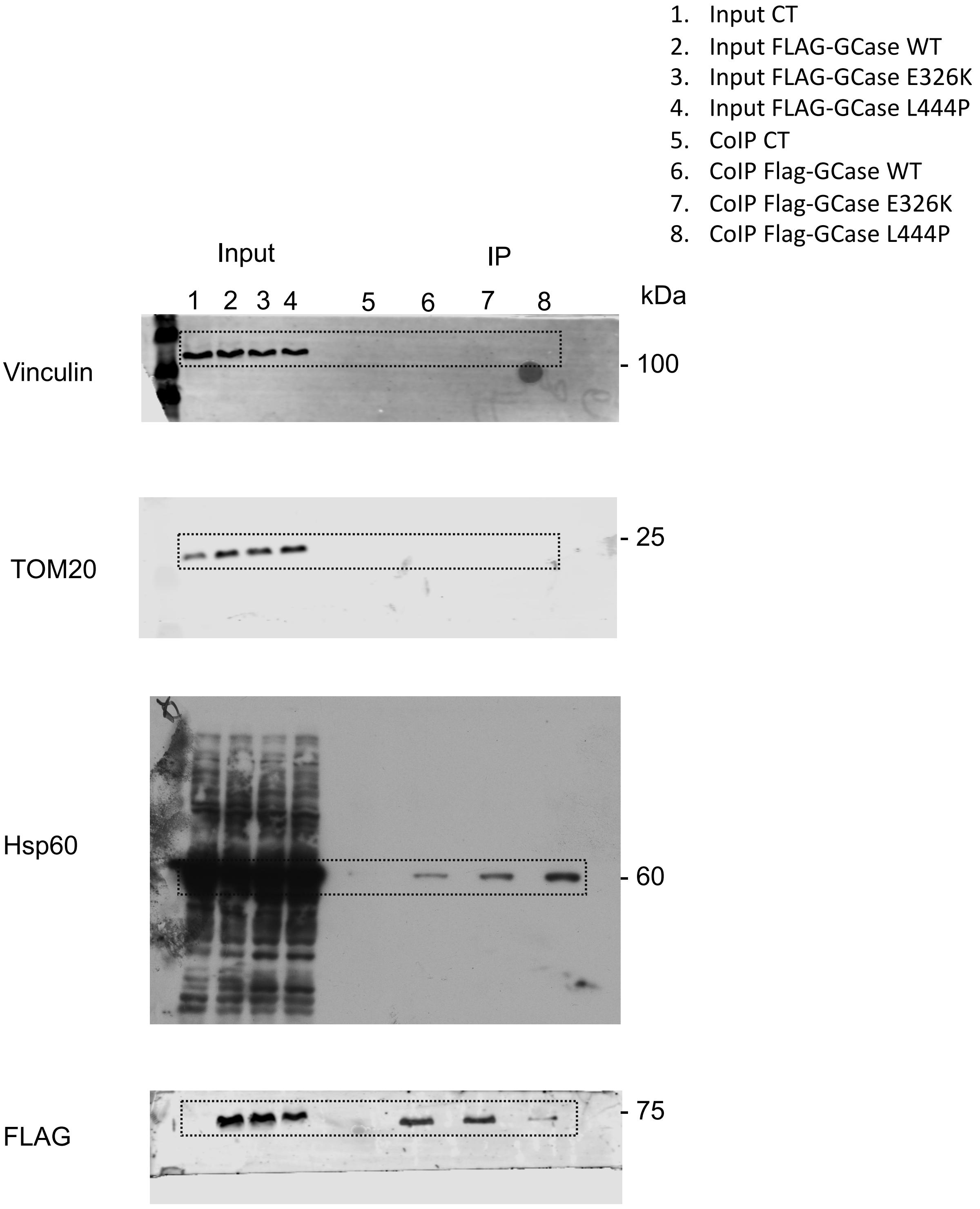

Supplementary Figure 3C

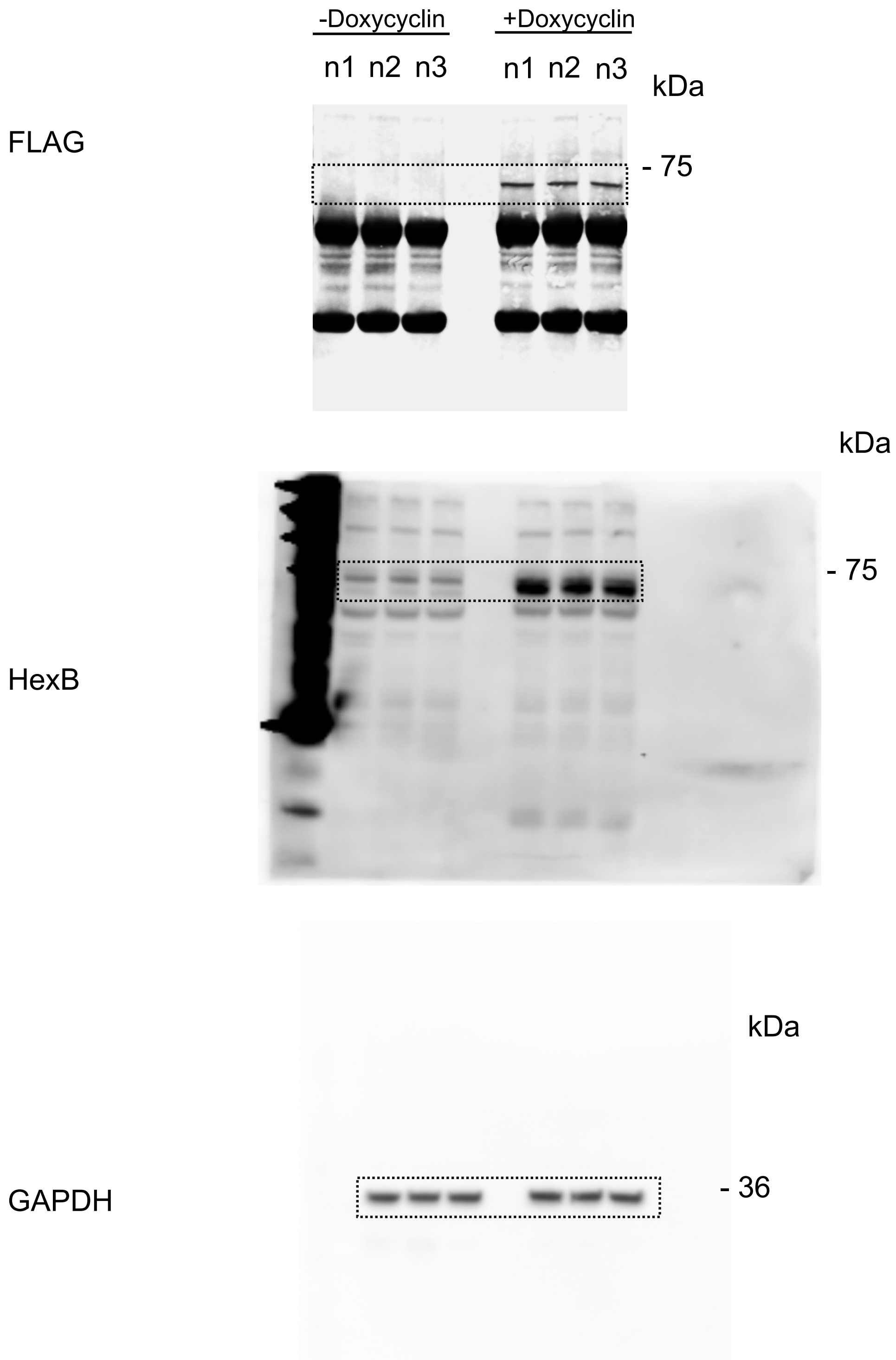

Supplementary Figure 3E

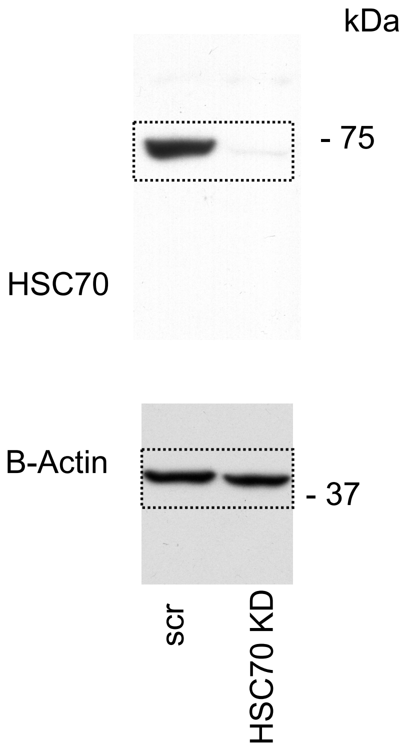

Supplementary Figure 3G

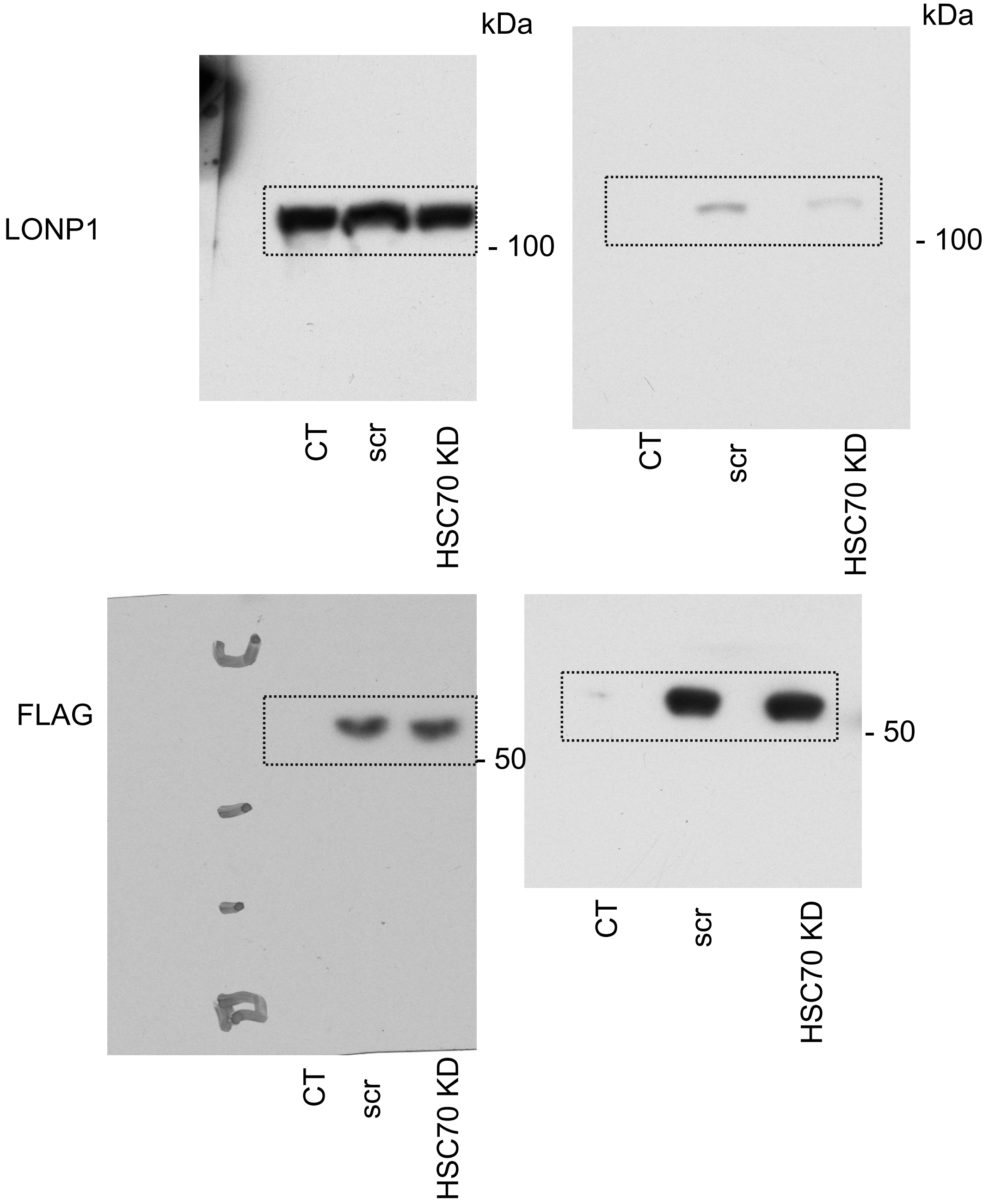

Supplementary Figure 3H

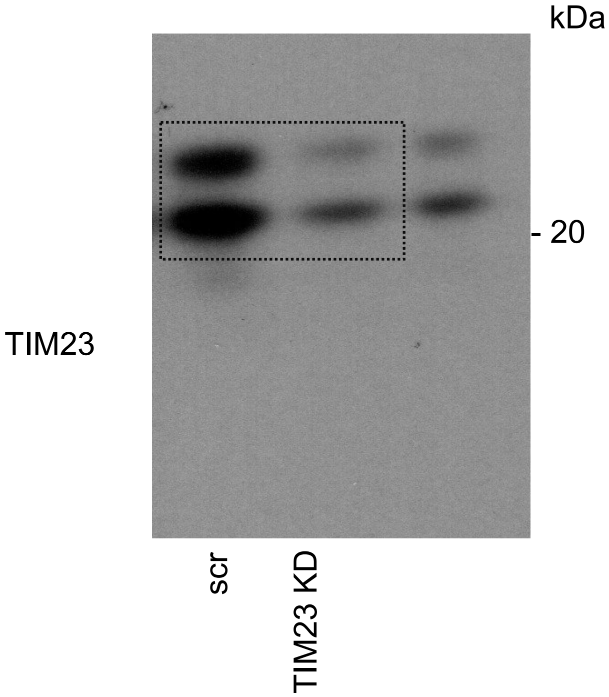

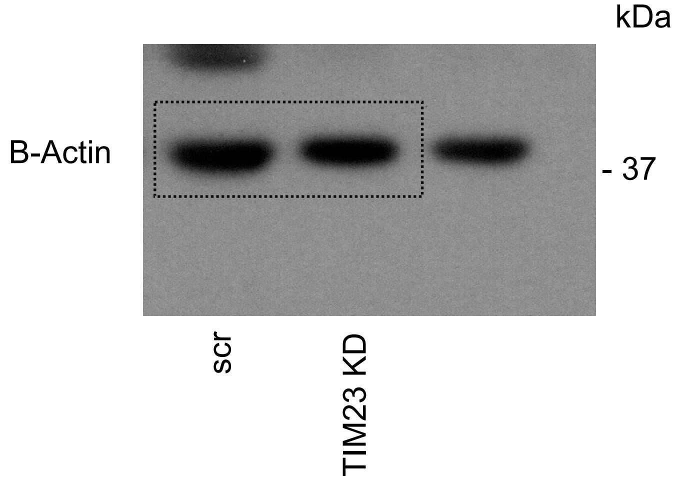

Supplementary Figure 3I

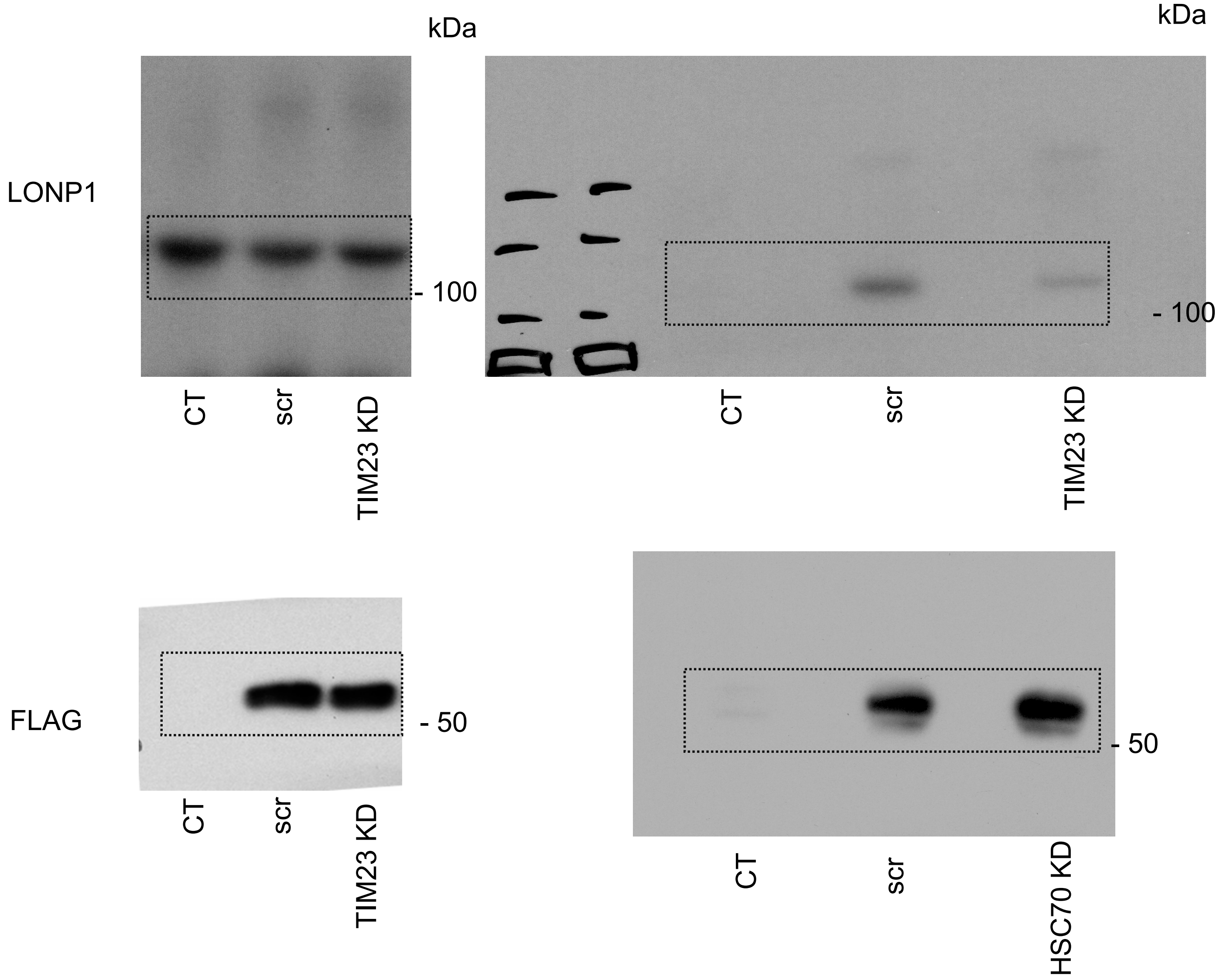

Supplementary Figure 3J

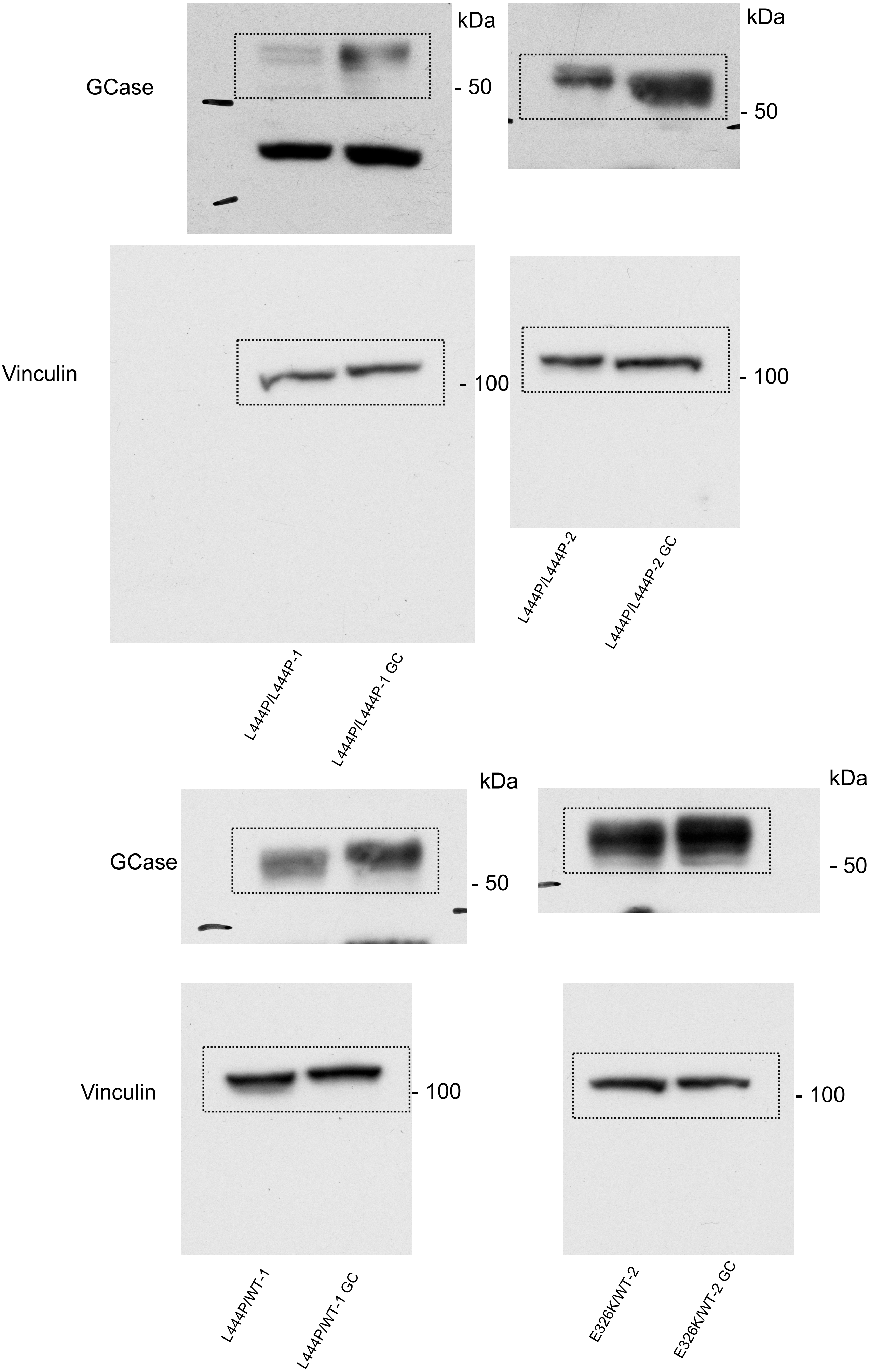

Supplementary Figure 4C

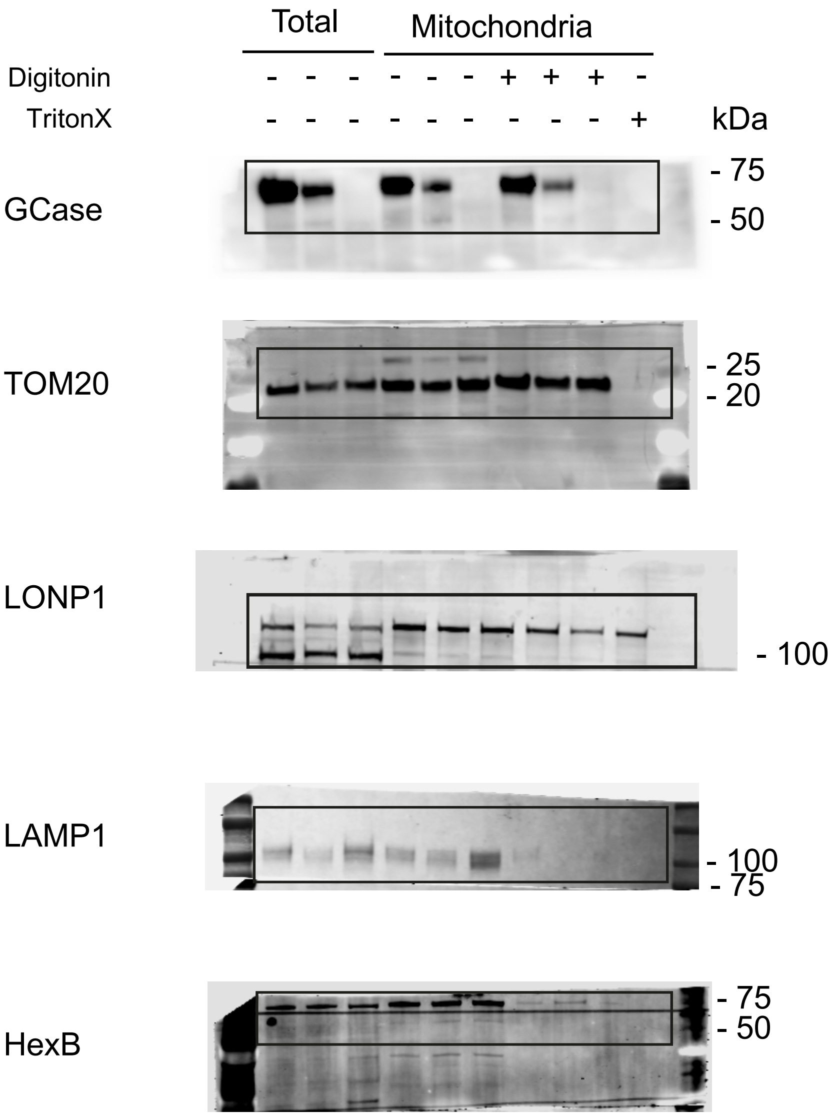

Supplementary Figure 4D

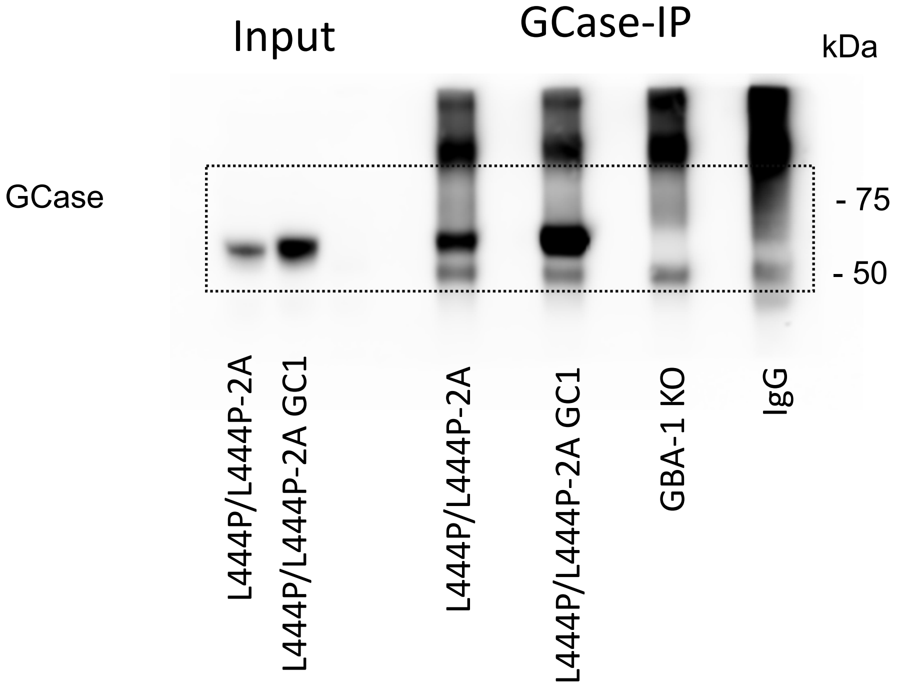

Supplementary Figure 4E

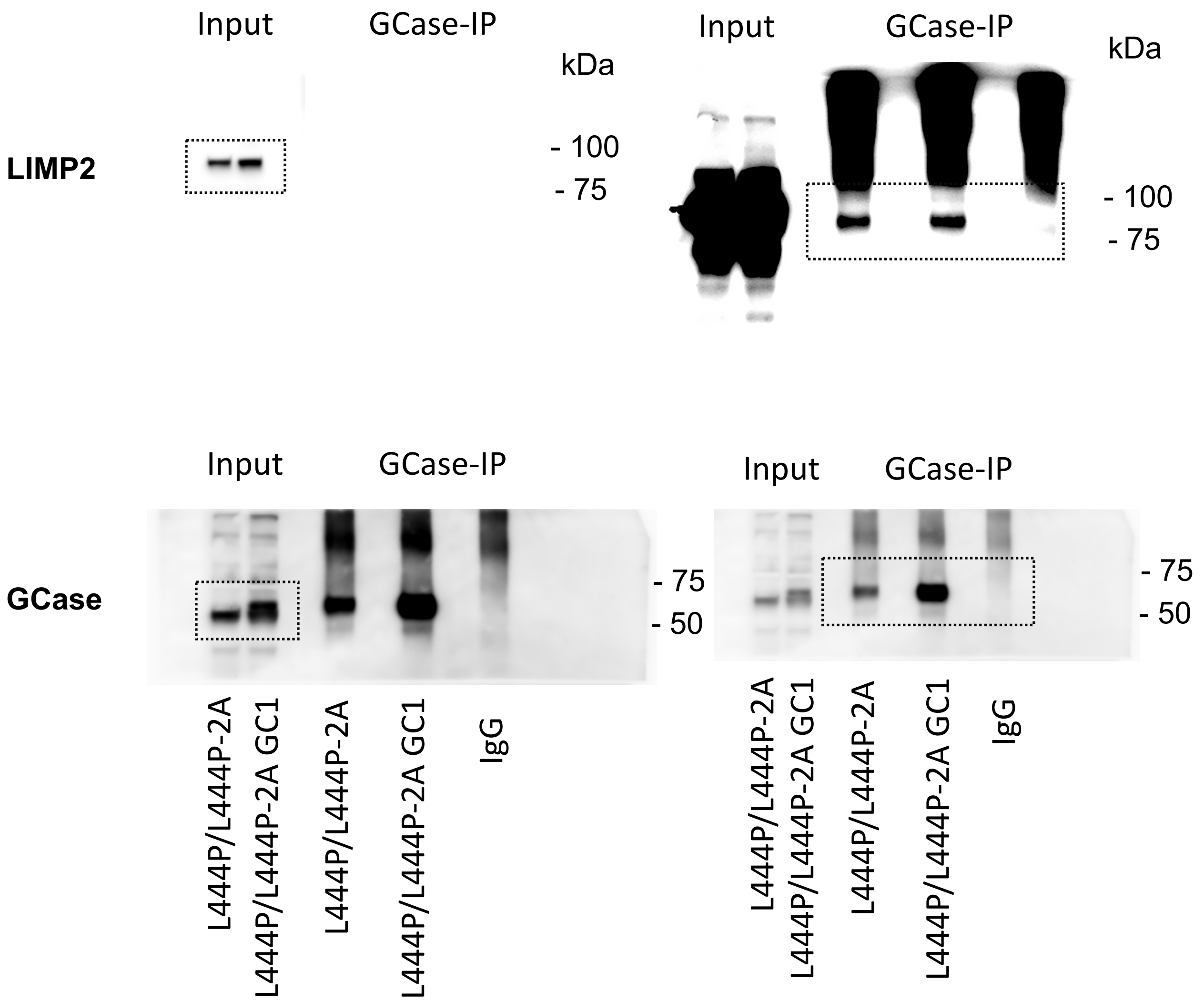

Supplementary Figure 4F

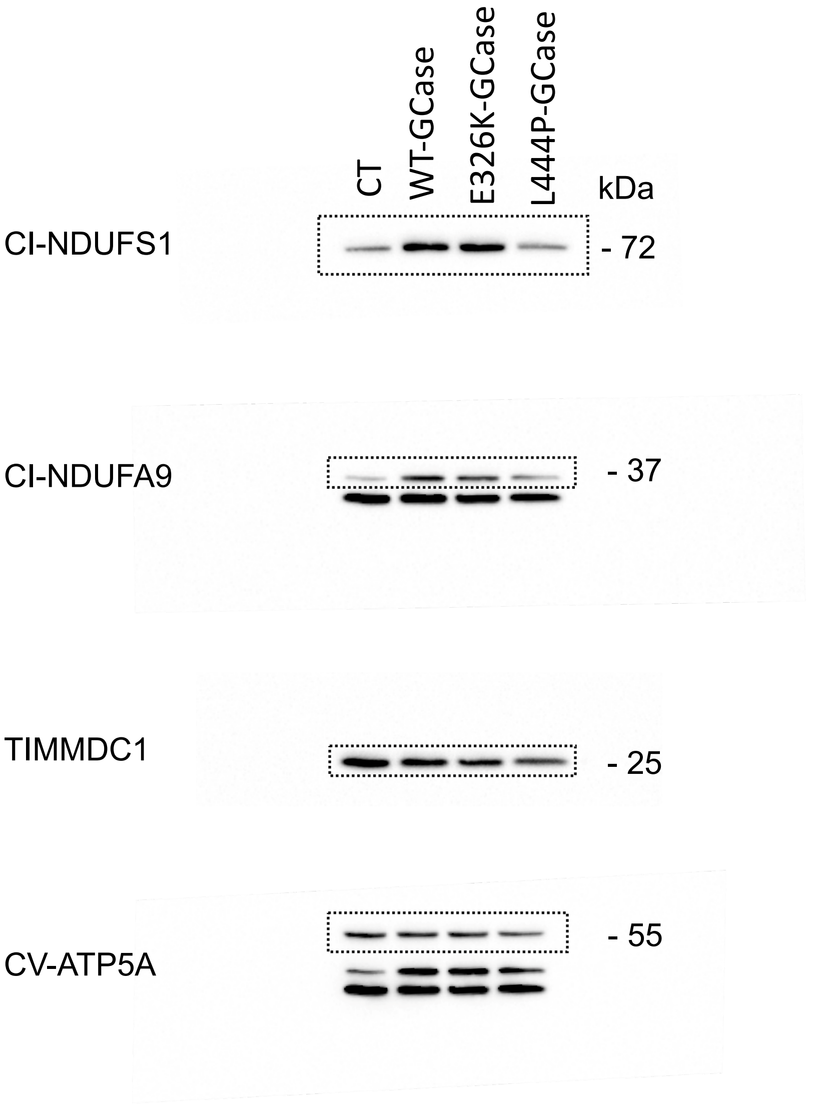

Supplementary Figure 6A

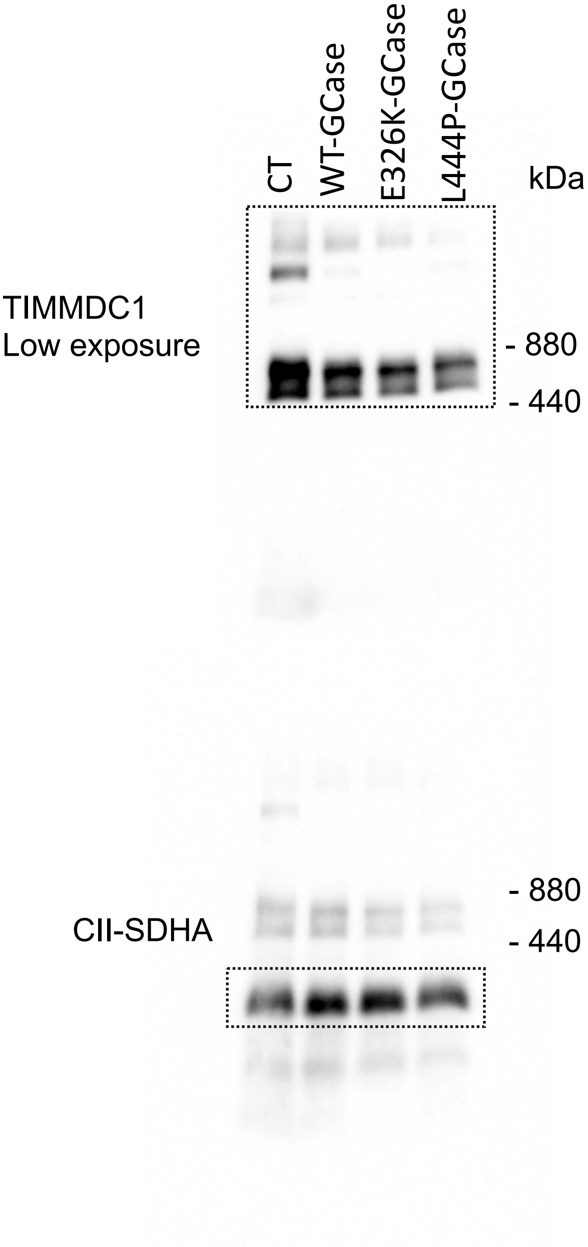

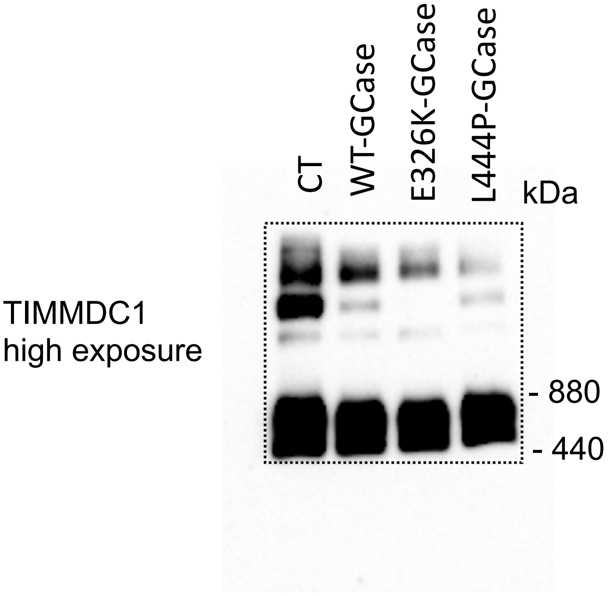

Supplementary Figure 6B

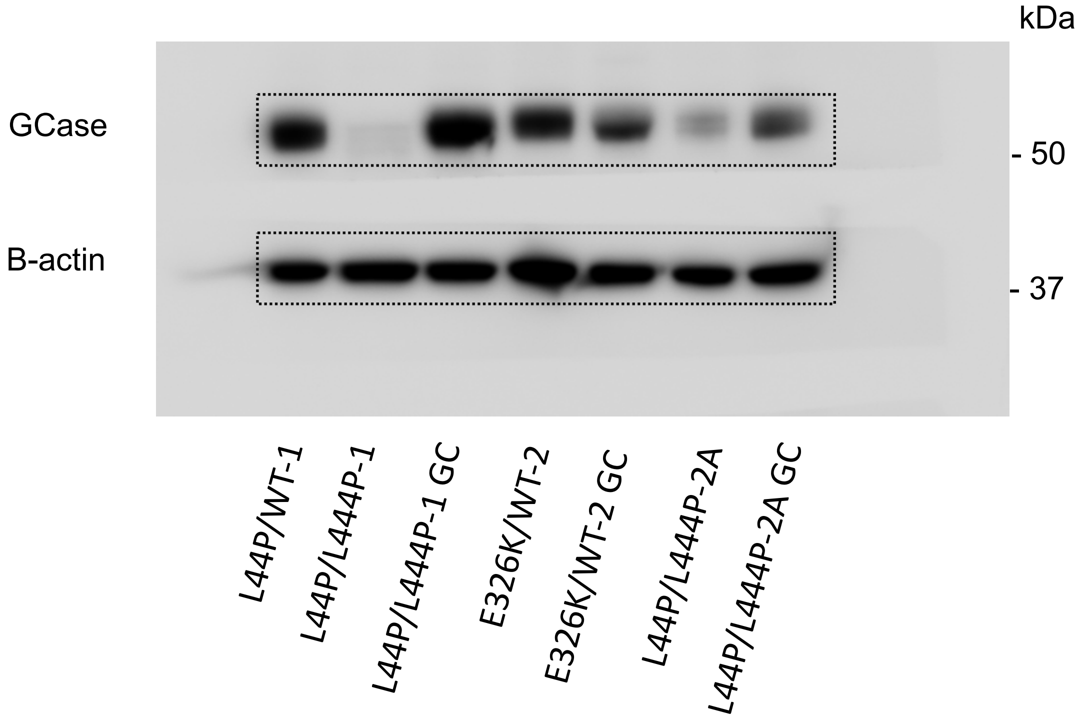

Supplementary Figure 9B

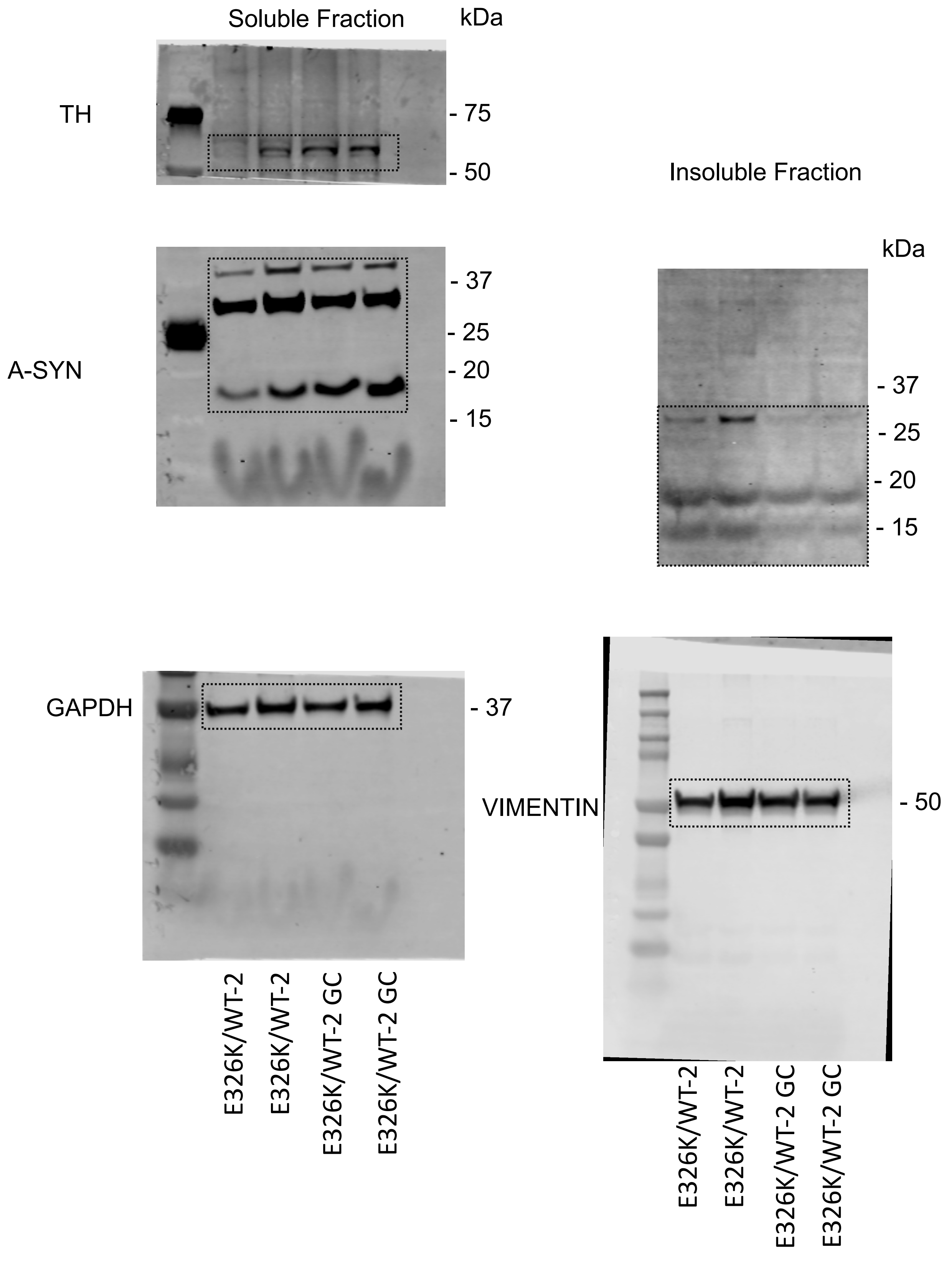

Supplementary Figure 9C
